# The effect of large amplitude vibration on the pressure-dependent absorption of a structure multiple cavity system

**DOI:** 10.1371/journal.pone.0219257

**Published:** 2019-07-09

**Authors:** Yiu-Yin Lee

**Affiliations:** Department of Architecture and Civil Engineering, City University of Hong Kong, Kowloon Tong, Kowloon, Hong Kong SAR, China; Universiti Sains Malaysia, MALAYSIA

## Abstract

This study addresses the effects of large-amplitude vibration on the pressure-dependent absorption of a structure multiple-cavity system. It is the first study to consider the effects of large-amplitude vibration and pressure-dependent absorption. Previous studies considered only one of these two factors in the absorption calculation of a perforated panel absorber. Nonlinear differential equations, which represent the structural vibration of a perforated panel absorber, are coupled with the wave equation, which represents the acoustic pressures induced within the cavities. The coupled nonlinear differential equations are solved with the proposed harmonic balance method, which has recently been adopted to solve nonlinear beam problems and other nonlinear structural-acoustic problems. Its main advantage is that when compared with the classical harmonic balance method, the proposed method generates fewer nonlinear algebraic equations during the solution process. In addition, the solution form of the nonlinear differential equations from this classical method can be expressed in terms of a set of symbolic parameters with various physical meanings. If a numerical method is used, there is no analytic solution form, and the final solution is a set of numerical values. The effects of the excitation magnitude, cavity depth, perforation ratio, and hole diameter on the sound absorption of a panel absorber are investigated, and mode and solution convergence studies are also performed. The solutions from the proposed harmonic balance method and a numerical integration method are compared. The numerical results show that the present harmonic balance solutions agree reasonably well with those obtained with the numerical integration method. Several important observations can be made. First, perforation nonlinearity is a very important factor in the absorption of a panel absorber at the off structural resonant frequency range. The settings of the hole diameter, perforation ratio, and cavity depth for optimal absorption differ greatly with consideration of perforation nonlinearity. Second, the “jump up phenomenon,” which does not occur in the case of linear perforation, is observed when perforation nonlinearity is considered. Third, one or more absorption troughs, which worsen the average absorption performance, may exist in cases with multiple cavities.

## 1 Introduction

In recent decades, many researchers have investigated the absorption properties of various perforated panel absorbers (e.g. [1–7]). Most of their studies considered sound absorption independent of the excitation pressure. In practice, a panel absorber is subject to external excitations and is forced to vibrate. Some studies have considered the linear panel vibration effect and the sound absorption independent of the excitation pressure. However, a panel absorber is normally a thin sheet, and its vibration amplitude is quite large. In some situations, high-magnitude excitations are imposed on the panel surface to make the perforation effect quite nonlinear. For example, Kang and Fuchs [8] studied the absorption of open-weave textiles and microperforated membranes backed by air. They presented a theoretical method to predict the absorption of such structures and demonstrated that their absorption performance could be very high. With appropriate parameters, the absorption coefficient of a glass-fiber textile or a microperforated membrane mounted 100 mm from a rigid wall could exceed 0.4 over three to four octaves. Sakagami et al. [9] investigated the sound absorption of a double-leaf microperforated panel with an air-back cavity and a rigid-back wall. They compared the absorption solutions from electro-acoustical equivalent circuit analysis and the Helmholtz-Kirchhoff integral formulation. Their comparison showed that the difference occurred mainly around the resonant frequency ranges. Toyoda and Takahashi [10] developed a model of sound radiation from an infinite plate with an absorptive facing and found that the acoustic radiation depended on the change in impedance and the absorption coefficient, which could thus be a way of reducing the radiation from the vibrating surface via the selection of an appropriate impedance surface. Lee et al. [11] derived a numerical model of the acoustic impedance of perforated plates under bias flow conditions with consideration of the interaction effect between orifices. They observed that the reactance decreased as the porosity increased because the attached mass on the orifices was reduced by the interaction effect. The result from the new model, which considered the interaction effect, agreed well with the experimental data. Park [12] proposed a design method of microperforated panel absorbers for enhancement of acoustic absorption inside the payload fairings of launch vehicles. The developed design charts to mitigate acoustic loads in launcher fairings and demonstrated that a microperforated panel absorber with a large hole diameter could achieve good absorption only with a high excitation level. Chiang and Choy [13] investigated the acoustic behaviors of the microperforated panel absorber array in a nonlinear regime with moderate acoustic pressure excitation. The absorber array was constructed by three parallel-arranged absorbers with different cavity depths. It was found that that absorption of the absorber array was affected by the incident sound pressure when the excitation level exceeded 100 dB. With appropriate selection of the absorber’s configurations, the absorber array in a nonlinear regime could achieve better absorption performance than one in a linear regime because the improvement in equivalent acoustic impedance could match with the ambient air over a wide frequency range.

Moreover, vibro-acoustic/fluid-structure modeling has been a hot research topic for many years. Various techniques have been developed to solve panel-cavity problems (e.g. [14–21]). These studies adopted the assumption of linear vibration or small-amplitude vibration. For example, Pan et al. [22] performed an analysis of the low-frequency acoustic response in a damped rectangular enclosure. The acoustic properties of an enclosure were modelled with the modal expansion approach. The results showed that a frequency-dependent modal parameter could be used to describe the acoustic response. The modal parameter was defined as the specific acoustic modal admittance, which described the contribution of all boundaries (locally and modally reactive and air leakage) to the modal damping and the shift of natural frequencies. Cui et al. [23] proposed a solution procedure that focused on the accurate and efficient numerical implementation of acoustic-structure coupling formulations using the edge-based smoothed finite-element method (the coupled ES/GW-FEM formulation) for the flexible shell and the gradient-weighted finite-element method for the acoustic fluid field. They developed the coupled ES/GW-FEM formulation and found that it could achieve much greater accuracy and greater reliability than coupled FEM/FEM in solving practical engineering problems. Wang et al. [24] developed a coupled smoothed finite-element method (S-FEM) for structural-acoustic analysis of shells. A gradient-smoothing technique was introduced to perform the strain-smoothing operation. The discretized system equations were obtained using the smoothed Galerkin weak form, and the numerical integration was then applied over the further formed edge-based and face-based smoothing domains, respectively. From the numerical results, the effectiveness and accuracy of the coupled S-FEM were verified for the structural-acoustic problems. The comparisons showed that the difference between the results from the proposed method and those of the other method were less than 5.6%. Chen et al. [25] studied the vibrational behavior and far-field sound radiation of a submerged stiffened conical shell at low frequencies. A smeared approach was adopted to model the ring stiffeners. The dynamic response solution was expressed in the terms of power series. The far-field sound pressure was computed with the element radiation superposition method. The proposed method was also validated with the numerical results.

In a problem of nonlinear panel-cavity, a solution method must be required to solve the nonlinear differential equations (e.g. [26–29]). In fact, many previous studies [30–37] included various techniques developed to solve problems of nonlinear vibrations or oscillations that are useful for solving nonlinear panel-cavity problems. For example, Huang et al. [34] presented the Precise Hsu’s method to analyze the stability of periodic solutions of multiple-degrees-of-freedom systems with cubic nonlinearity. They adopted the incremental harmonic balance method to obtain the solution of nonlinear vibration differential equations. Hsu’s method was then adopted to compute the transition matrix at the end of one period. The proposed method was verified by a numerical integration method. In the comparisons, the difference between the results from the proposed method and other method was less than 1%. Chen et al. [35] extended the multidimensional Lindstedt-Poincare (MDLP) method to the nonlinear vibration analysis of axially moving systems. The forced response of an axially moving beam was studied with internal resonance between the first two transverse modes. The numerical results showed that the MDLP method would be a straightforward and efficient method; and the results from the MDLP method could agree reasonably well with those obtained with the incremental harmonic balance method. Guo et al. [36] presented a simple and rigorous solution procedure of residual harmonic balance for certain autonomous ordinary differential systems. Three kinds of differential equations that involve general, fractional, and delay ordinary differential systems were considered in the numerical cases. The results from the proposed method could match well the exact solutions or numerical solutions for a wide range of control parameters. The residual harmonic balance solution procedure proved to be very effective for these autonomous differential systems. Hasan et al. [37] proposed the multilevel residue harmonic balance method for nonlinear vibrations of multimode flexible beams on an elastic foundation subject to external harmonic excitation. Only one set of nonlinear algebraic equations was generated in the procedures, and the computation effort would be much less.

As mentioned above, this study is the first to consider the effects of large-amplitude vibration and pressure-dependent absorption. The corresponding nonlinear formations and governing equations are developed and solved with the multilevel residue harmonic balance method. The effects of the excitation magnitude, cavity depth, perforation ratio, and hole diameter on the sound absorption of a panel absorber are investigated.

## 2 Theory

**Fig 1:**
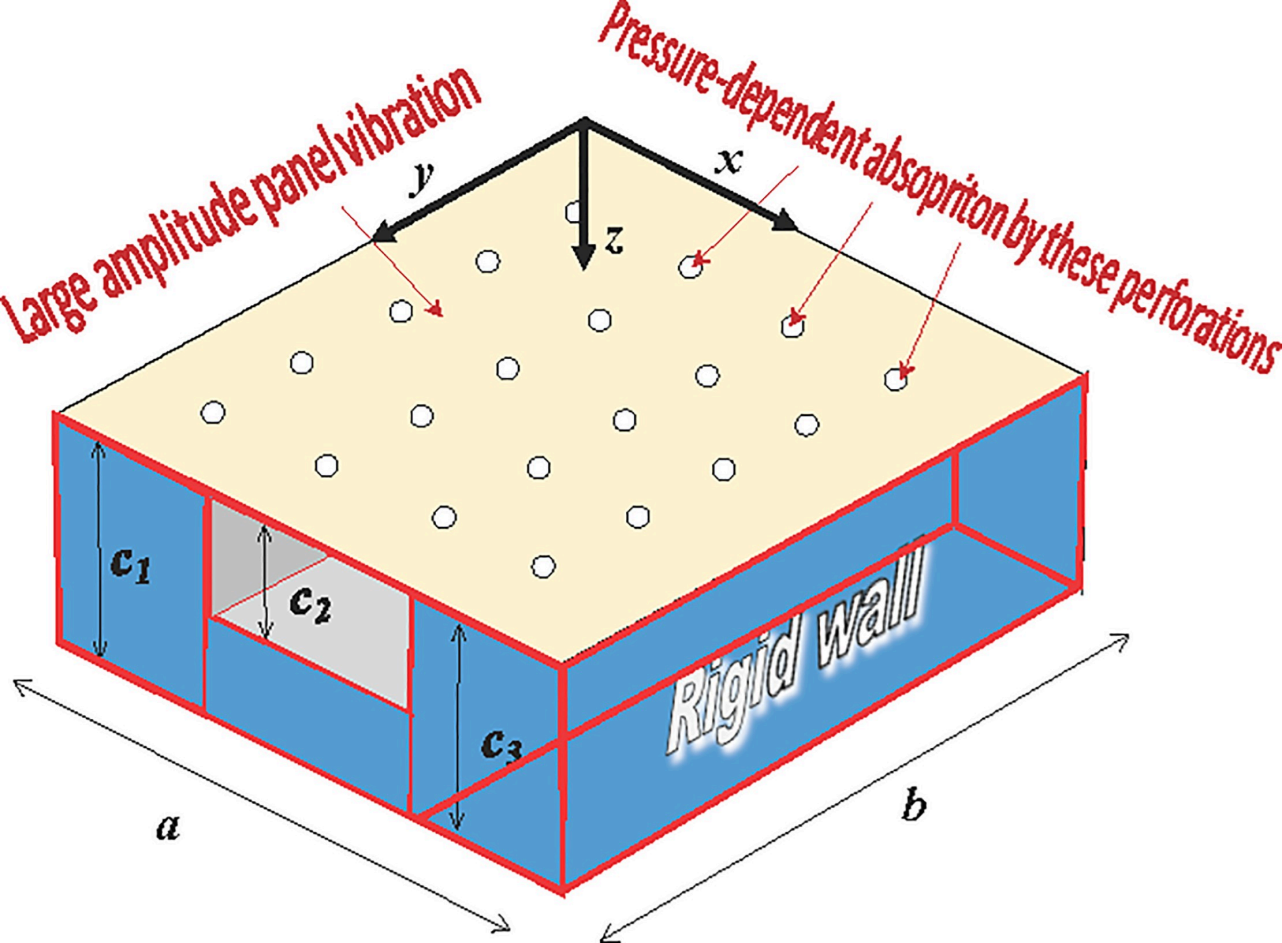
A perforated panel absorber with multiple cavities subject to the effect of large amplitude vibration.

Fig 1 shows a nonlinear perforated panel backed by multiple cavities. The pressure field can be computed with the following homogenous wave equation (e.g. [2])

$$\nabla^{2}P-\frac{1}{C_{a}^{2}}\frac{\partial^{2}P}{\partial t^{2}}=0$$ (1)

where *P* is the acoustic pressure induced by the panel vibration and *C*_*a*_ is the sound speed.

In the case of a single cavity, the cavity is formed by one flexible perforated panel and five rigid panels. In the case of three cavities, the cavity is internally divided into three small rectangular cavities. The two internal partition panels are also rigid. The vibrations and displacements of the rigid panels are zero, so the first derivative of the pressure on each of these rigid panels is zero. The first derivative of the pressure on the flexible panel is proportional to its acceleration. The acoustic boundary conditions are listed in the following equations.

*for a single cavity*,

$$\frac{\partial P}{\partial x}=0\;\mathrm{at}\;x=0\;\mathrm{or}\;a$$ (2)

or *a*

$$\frac{\partial P}{\partial z}=0\;\mathrm{at}\;z=c_{1}$$ (3)

*for three cavities*,

$$\frac{\partial P}{\partial x}=0\;\mathrm{at}\;x=0\;\mathrm{or}\;a/3\;\mathrm{or}\;2a/3\;\mathrm{or}\;a$$ (4)

$$\frac{\partial P}{\partial z}=0\;\mathrm{at}\;z=c_{1},\;x=0\;\mathrm{to}\;a/3$$ (5)

$$\frac{\partial P}{\partial z}=0\;\mathrm{at}\;z=c_{2},\;x=a/3\;\mathrm{to}\;2\;a/3$$ (6)

$$\frac{\partial P}{\partial z}=0\;\mathrm{at}\;z=c_{3},\;x=2\;a/3\;\mathrm{to}\;a$$ (7)

*for a single cavity or three cavities*

$$\frac{\partial P}{\partial y}=0\;\mathrm{at}\;y=0\;\mathrm{and}\;b$$ (8)

$$\frac{\partial P}{\partial z}=-\rho_{a}\frac{\partial^{2}W(x,y,t)}{\partial t^{2}}\;\mathrm{at}\;z=0$$ (9)

where *a*, *b*, *c*_*1*_, *c*_*2*_, and *c*_*3*_ are the panel width, length and cavity depths, respectively; *ρ*_*a*_ is the air density; and *W*(*x*,*y*,*t*) is the panel displacement response.

From the boundary conditions in Eqs (2–7), the acoustic cavity mode functions are given in the following,

*for a single cavity*,

$$\varphi_{uv}(x,y)=\cos\left(\frac{u\pi }{a}x\right)\cos\left(\frac{v\pi }{b}y\right)\;\mathrm{at}\;x=0\;\mathrm{to}\;a$$ (10)

*for three cavities*,

$$\varphi_{uv}\left(x,y\right)=\cos\left(\frac{u\pi }{\frac{a}{3}}x\right)\cos\left(\frac{v\pi }{b}y\right)\;\mathrm{at}\;x=0\;\mathrm{to}\;a/3$$ (11)

$$\varphi_{uv}\left(x,y\right)=\cos\left(\frac{u\pi }{\frac{a}{3}}\left(x-\frac{a}{3}\right)\right)\cos\left(\frac{v\pi }{b}y\right)\;\mathrm{at}\;x=a/3\;\mathrm{to}\;2a/3$$ (12)

$$\varphi_{uv}\left(x,y\right)=\cos\left(\frac{u\pi }{\frac{a}{3}}\left(x-\frac{2}{3}a\right)\right)\cos\left(\frac{v\pi }{b}y\right)\;\mathrm{at}\;x=2a/3\;\mathrm{to}\;a$$ (13)

where *u* and *v* are the acoustic mode numbers

By putting Eqs (10–13) into Eq (1), the general expression for the acoustic pressure in the three cases is given by [7,28]

$$P(x,y,z,t)=\rho_{a}\omega^{2}\sum_{u}^{U}\sum_{v}^{V}\frac{\cosh\left(\mu_{uv}z\right)}{\sinh\left(\mu_{uv}c\right)}\frac{A(t)\alpha_{uv}}{\mu_{uv}\alpha_{\varphi \varphi }}\varphi_{uv}(x,y)$$ (14)

where *ω* is the excitation frequency; *A(t)* is the modal response of the panel; *ρ*_*a*_ is the air density; *U* and *V* are the numbers of acoustic modes used; and

$$\alpha_{uv}=\int_{0}^{b}\int_{0}^{a}\varphi_{uv}\phi dxdy$$ (15)

$$\alpha_{\varphi \varphi }=\int_{0}^{b}\int_{0}^{a}\varphi_{uv}\varphi_{uv}dxdy$$ (16)

$$\phi =\sin\left(\frac{\pi x}{a}\right)\sin\left(\frac{\pi y}{b}\right)$$ (17)

$$\mu_{uv}=\frac{1}{C_{a}}\sqrt{\omega_{uv}^{2}-\omega^{2}}$$ (18)

where *ω*_*uv*_ is the acoustic resonant frequency of the (*u*,*v*) mode

The acoustic pressure in Eq (14) is then multiplied by the mode shape function, and integration is taken over the panel area to obtain the acoustic force upon the panel,

$$P_{c}=\rho_{a}\omega^{2}\sum_{u}^{U}\sum_{v}^{V}\frac{\coth\left(\mu_{uv}c\right)}{\mu_{uv}}\frac{|A|{\left(\alpha_{uv}\right)}^{2}}{\alpha_{\varphi \varphi }\phi_{\varphi \varphi }}$$ (19)

where |*A*| is the panel displacement amplitude; and

$$\alpha_{\phi \phi }=\int_{0}^{b}\int_{0}^{a}\phi \phi dxdy$$ (20)

The normalized cavity impedance is then defined by [7,38]

$$Z_{c}=\frac{P_{c}}{{\rho_{a}C_{a}\bar{V}}_{c}}$$ (21)

where

$${\bar{V}}_{c}=j\omega |A|$$ (22)

$$j=\sqrt{-1}$$ (23)

The normalized impedance of the large-amplitude vibrating panel is derived here. The governing equation of a large-amplitude vibrating panel subject to harmonic excitation is given by [7,28]

$$\rho \frac{d^{2}A}{dt^{2}}+\rho \omega_{o}^{2}A+\beta A^{3}=P_{e}sin(\omega t)$$ (24)

where

$$\omega_{o}=\sqrt{\frac{E\tau^{2}}{12\rho (1-\nu^{2})}}\left({\left(\frac{\pi }{a}\right)}^{2}+{\left(\frac{\pi }{b}\right)}^{2}\right),\;linear\;natural\;frequency$$ (25)

$$\beta =\frac{E\tau }{12(1-\nu^{2})}\frac{\gamma }{a^{4}},\;noninear\;natural\;frequnecy$$ (26)

$$\gamma =3\;\pi^{4}\left[(\frac{3}{4}-\frac{v^{2}}{4})(1+r^{2})+vr^{2}\right]$$ (27)

r=a/b,aspectratio (28)

$$P_{e}=\frac{\alpha_{\phi }}{\alpha_{\phi \phi }}F_{e}$$ (29)

$$F_{e}=\kappa \;\rho \tau g$$ (30)

$$\alpha_{\phi }=\int_{0}^{b}\int_{0}^{a}\phi dxdy$$ (31)

where *E = Young’s modulus; ν* = Poisson’s ratio; *ρ* = density per unit thickness; *τ* = panel thickness; *g* = gravity acceleration (9.81ms^-2^); and *κ* = dimensionless excitation parameter.

Eq (24) is then solved using the harmonic balance method in [31, 36–37]. The periodic solution form is given by

$$A(t)=A_{1,\sin}(t)+A_{3,\sin}(t)+A_{5,\sin}(t)$$ (32)

⇒

$$\begin{array}{r} A(t)=\varepsilon^{0}A0_{1}\sin(\omega t)+\varepsilon A1_{1}\sin(\omega t)+\varepsilon^{2}\;A2_{1}\sin(\omega t)+\\ +\varepsilon A1_{3}\sin(3\omega t)+\varepsilon^{2}A2_{3}\sin(3\omega t)+\\ +\varepsilon^{2}A2_{5}\sin(5\omega t) \end{array}$$ (33)

where

$$A0(t)={A0}_{1}sin(\omega t)$$ (34)

$$A1(t)={A1}_{1}sin(\omega t)+{A1}_{3}sin(3\omega t)$$ (35)

$$A2(t)={A2}_{1}sin(\omega t)+{A2}_{3}sin(3\omega t)+{A2}_{5}sin(5\omega t)$$ (36)

Note that *ε* is a perturbation parameter that is used to classify the level of each term. For example, if a term is associated with *ε*^2^, it is a second-level term.

According to [31, 36–37], Eq (32) is substituted into (24) and those terms associated with *ε*^*0*^, *ε*^*1*^, and *ε*^*2*^, respectively, are considered to set up the following nonlinear equations.

$$\Pi (\omega )A0_{1}+\frac{3}{4}\beta_{c}A0_{1}^{3}=P_{e}$$ (37)

$$\pi (\omega )A1_{1}\sin(\omega t)+\pi (3\omega )A1_{3}\sin(3\omega t)+3\beta_{c}{\left(A0\right)}^{2}A1+\Delta_{A0}=L_{1}$$ (38)

$$\begin{array}{l} \pi (\omega )A2_{1}\sin(\omega t)+\pi (3\omega )A2_{3}\sin(3\omega t)+\pi (5\omega )A2_{5}\sin(5\omega t)+3\beta_{c}A0{\left(A1\right)}^{2}+3\beta_{c}A2{\left(A0\right)}^{2}\\ \;+\Delta_{A1}=L_{2} \end{array}$$ (39)

where *L*_1_ and *L*_2_ represent the sums of the first-level and second-level terms. The unbalanced residual in the first level;

$$\Pi (\omega )=\rho_{c}\left(-\omega^{2}+\omega_{c}^{2}\right),\;\mathrm{for}\;\mathrm{undamped}\;\mathrm{panel}$$ (40)

$$\Pi (\omega )=\rho_{c}\left(-\omega^{2}+\omega_{c}^{2}\right)+j\xi \omega \omega_{p},\;\mathrm{for}\;\mathrm{damped}\;\mathrm{panel}$$ (41)

$$\Delta_{A0}=-\frac{1}{4}\beta_{c}{A0}_{1}^{3}sin(3\omega t)$$ (42)

$$\Delta_{A1}=\frac{\int_{0}^{2\pi }{L1}_{c}sin(5\omega t)dt}{\int_{0}^{2\pi }(sin(5\omega t){)}^{2}dt}sin(5\omega t)$$ (43)

where *ω*_*c*_ and *ω*_*p*_ are the linear resonant panel frequency and the peak frequency, respectively.

*A0*_*1*_ can be found directly from Eq (37), and *A1*_*1*_, *A1*_*3*_, *A2*_*1*_, *A2*_*3*_, and *A2*_*5*_ in Eqs (38 and 39) can be found by solving the following harmonic balance equations

$$\int_{0}^{2\pi }L_{1}\;sin(\omega t)dt=0$$ (44)

$$\int_{0}^{2\pi }L_{1}\;sin(3\omega t)dt=0$$ (45)

$$\int_{0}^{2\pi }L_{2}\;sin(\omega t)dt=0$$ (46)

$$\int_{0}^{2\pi }L_{2}\;sin(3\omega t)dt=0$$ (47)

$$\int_{0}^{2\pi }L_{2}\;sin(5\omega t)dt=0$$ (48)

Similarly, the normalized nonlinear impedance is defined by [7,38]

$$Z_{p}=\frac{P_{e}}{{\rho_{a}C_{a}\bar{\bar{V}}}_{p}}$$ (49)

where the complex form of the *h*^*th*^ harmonic velocity amplitude and overall velocity amplitude are defined by

$${\bar{V}}_{p}=\sum_{h=1,3,5\ldots }^{H}V_{p,h}$$ (50)

$$V_{p,h}=h\omega A_{h}e^{j(\frac{\pi }{2}+\theta_{h})}$$ (51)

where *θ*_*h*_ is the phase angle of the *h*^*th*^ harmonic component, and *A*_*h*_ is the amplitude of the *h*^*th*^ harmonic component and defined by

$$A_{5}=A2_{5},\mathrm{for}\;\mathrm{the}\;5^{\mathrm{th}}\;\mathrm{harmonic}\;\mathrm{component}$$ (52)

$$A_{3}=A1_{3}+A2_{3},\mathrm{for}\;\mathrm{the}\;3^{rd}\;\mathrm{harmonic}\;\mathrm{component}$$ (53)

$$A_{1}=A0_{1}+A1_{1}+A2_{1},\mathrm{for}\;\mathrm{the}\;1^{\mathrm{st}}\;\mathrm{harmonic}\;\mathrm{component}$$ (54)

The normalized impedance of the nonlinear perforation is derived here. According to Maa [2], the real and imaginary parts of the normalized impedance of linear perforation are given by

$$Z_{M,R}=0.147\frac{\tau }{\sigma \delta^{2}}\left(\sqrt{9+\frac{100\delta^{2}}{32}\frac{\omega }{2\pi }}+1.768\sqrt{\frac{\omega }{2\pi }}\frac{\delta^{2}}{\tau }\right)$$ (55)

$$Z_{M,I}=1.847\frac{\omega }{2\pi }\frac{\tau }{\sigma }\left(1+\frac{1}{\sqrt{9+50\delta^{2}\frac{\omega }{2\pi }}}+0.85\frac{\delta }{\tau }\right)$$ (56)

where *τ* is panel thickness, *σ* is the perforation ratio, and *δ* is the hole.

According to [12–13], the real and imaginary parts of the normalized impedance of nonlinear perforation are given by

$$Z_{M,R}=0.147\frac{\tau }{\sigma \delta^{2}}\left(\sqrt{9+\frac{100\delta^{2}}{32}\frac{\omega }{2\pi }}+1.768\sqrt{\frac{\omega }{2\pi }}\frac{\delta^{2}}{\tau }\right)(1+\lambda_{R})$$ (57)

$$Z_{M,I}=1.847\frac{\omega }{2\pi }\frac{\tau }{\sigma }\left(1+\frac{1}{\sqrt{9+50\delta^{2}\frac{\omega }{2\pi }}}+0.85\frac{\delta }{\tau }(1+\lambda_{I})\right)$$ (58)

where *λ*_*R*_ and *λ*_*I*_ are the terms due to the nonlinear perforation and given by

$$\lambda_{R}=\frac{1.59{\left(\frac{\delta }{\tau }\right)}^{0.06}\sigma^{-0.845}[\sigma (\sqrt{0.25+\frac{2P_{e}}{\rho_{a}c_{a}^{2}\sigma^{2}}}-0.5)]}{\frac{32\eta \tau }{\sigma \rho_{a}c_{a}\delta^{2}}[{\left(1+\frac{K^{2}}{32}\right)}^{1/2}+\frac{\sqrt{2}}{8}K\frac{\delta }{\tau }]}$$ (59)

$$\lambda_{I}=-\frac{\frac{1}{1-\sigma^{2}}(\sqrt{0.25+\frac{2P_{e}}{\rho_{a}c_{a}^{2}}\frac{1-\sigma^{2}}{\sigma^{2}}}-0.5)}{1+\frac{1}{1-\sigma^{2}}(\sqrt{0.25+\frac{2P_{e}}{\rho_{a}c_{a}^{2}}\frac{1-\sigma^{2}}{\sigma^{2}}}-0.5)}$$ (60)

where *η* is the coefficient of dynamic viscosity of air

Note that the normalized impedance of the nonlinear perforation is

$$Z_{M}=Z_{M,R}+iZ_{M,I}$$ (61)

According to [7,38], the overall impedance and absorption of a panel absorber subject to the effects of nonlinear perforation and large-amplitude vibration are given by

$$Z_{o}=\frac{Z_{M}Z_{p}}{Z_{M}+Z_{p}}+Z_{c}$$ (62)

$$\alpha_{o}=\frac{4Re(Z_{o})}{{\left(1+Re(Z_{o})\right)}^{2}+{\left(Im(Z_{o})\right)}^{2}}$$ (63)

## 3 Results and discussion

In this section, the material properties of the panel absorbers in all cases are listed as follows:

$$Young’s\;modulus=7.1\times 10^{10}\;N/m^{2};\;Poisson’s\;ratio=0.3;\;mass\;density=2700\;kg/m^{3}$$

**Fig 2:**
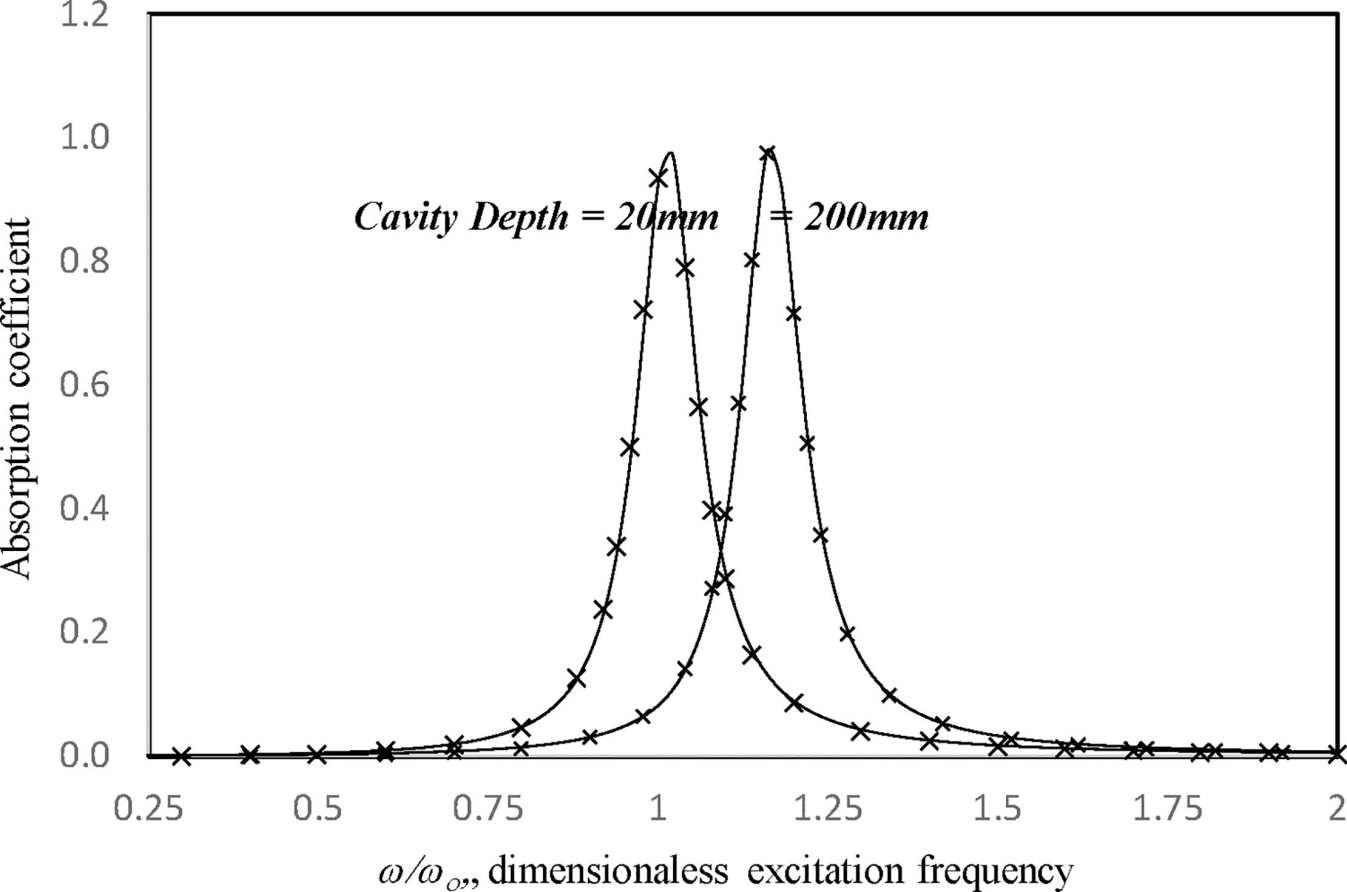
Comparison of the absorption results from the proposed harmonic balance method and numerical integration method [16] (*τ* = 2 mm, *a = b* = 0.2m, *κ* = 1, *ξ* = 0.03, single cavity, no perforation).

**Fig 3:**
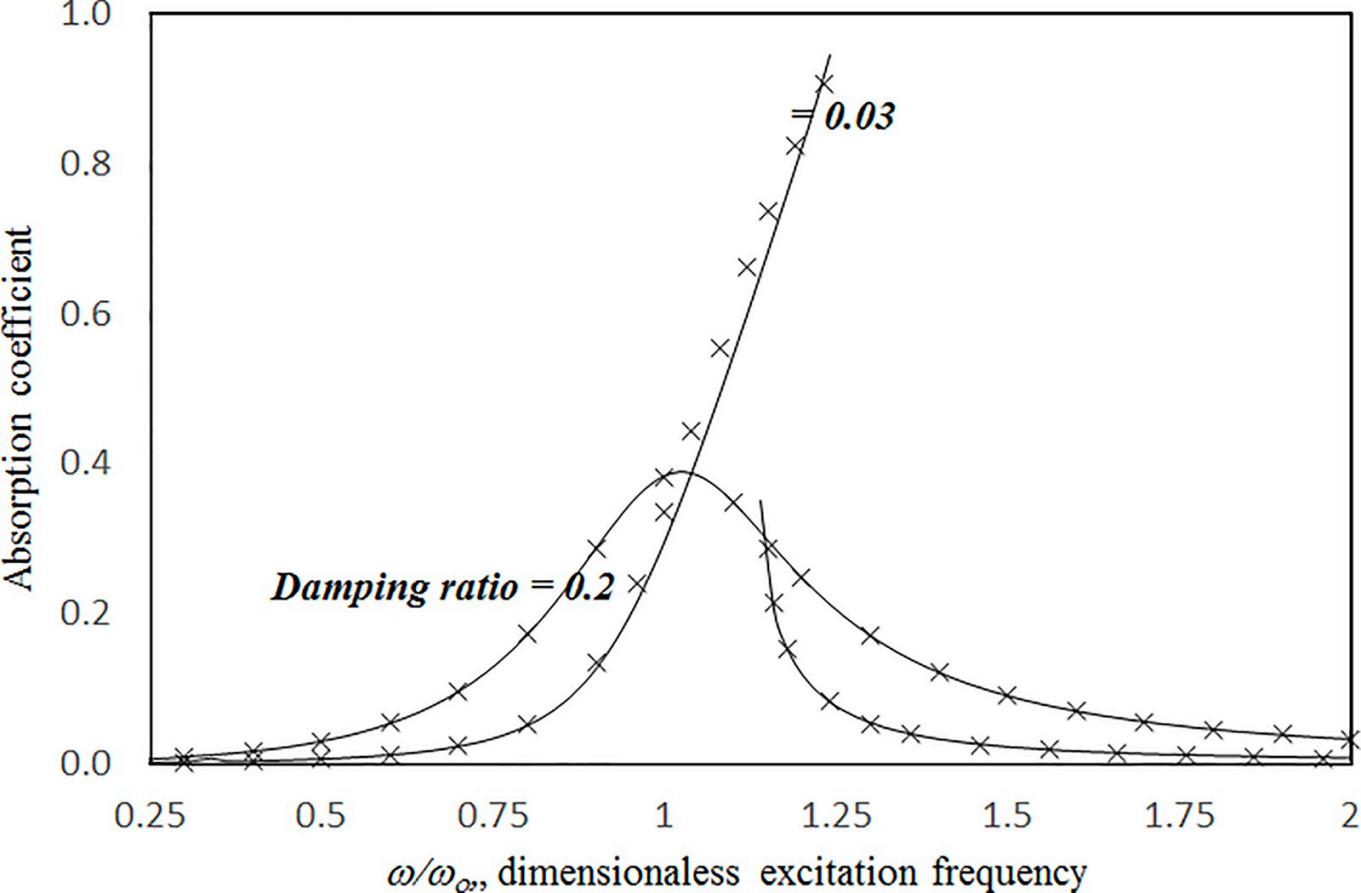
Comparison of the absorption results from the proposed harmonic balance method and numerical integration method [16] (*τ* = 2 mm, *a = b* = *c* = 0.2m, *κ* = 20, single cavity, no perforation).

**Fig 4:**
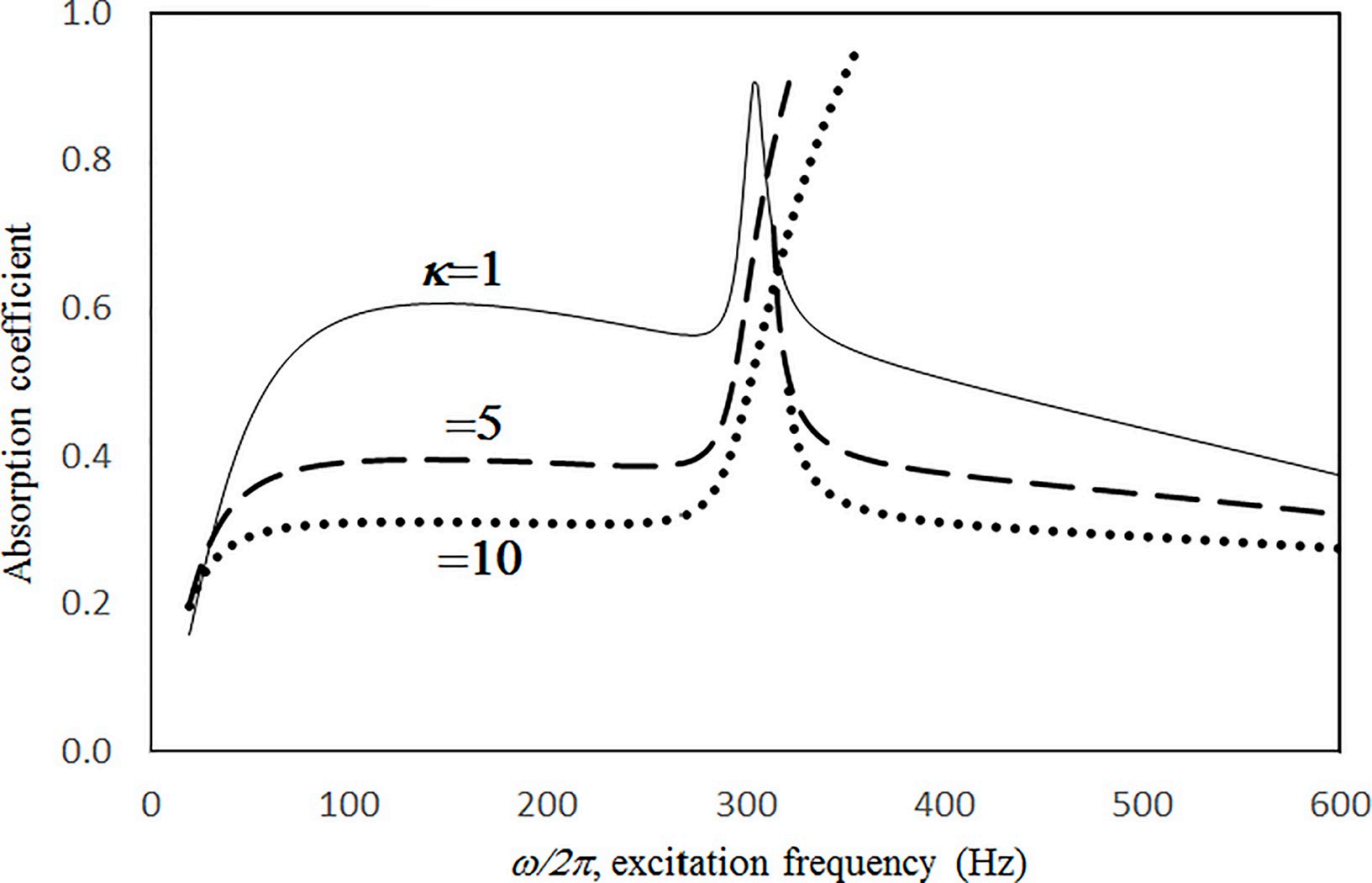
Absorption coefficient versus excitation frequency for various excitation magnitudes (*τ* = 2.5mm, *a = b = c =* 0.2m, *ξ* = 0.01, *σ =* 1%, *δ* = 0.5mm, single cavity, nonlinear perforation and large amplitude vibration).

In Tables 1–6, the convergence studies concern the absorptions of a large-amplitude vibrating panel absorber backed by a single cavity (no perforation effect) for various excitation levels and frequencies. The panel dimensions are 0.2 m × 0.2 m × 2 mm. The cavity depth is 0.1 m. The 1-, 4-, and 9-mode solutions and the zero, first, and second solutions are presented. Normally, for higher excitation or excitation frequency near the resonant frequency, more modes and higher-level solutions are needed to achieve acceptable accuracy. It can be seen from the six tables that the absorption values range from less than 0.05 to no more than 0.94. The maximum difference between the first-level and second-level solutions and the maximum difference between the 4- and 9-mode solutions are very small—less than 0.1%. Thus, the first-level and four-mode approach is used in the parametric studies. Figs 2 and 3 show the absorption coefficients of large-amplitude vibration plotted against the excitation frequency for cases with cavity depths *c* = 0.02 m and 0.2 m, damping ratios *ξ* = 0.2 and 0.03, and excitation parameters *κ* = 1 and 20. The results from the proposed harmonic balance method are compared with those from the numerical integration method adopted in [16]. In general, the results obtained from the two methods show good agreement. In the case of *κ =* 20, *ξ* = 0.03, and *c =* 0.2 m in Fig 4, some differences can be seen around the nonlinear resonant range from 1 to 1.25 *ω*_*c*_. In other nearly linear cases, the two solutions are almost identical, including those at the peak values.

**Table 1:** Absorption coefficient convergence for various numbers of modes used (*ω* = 0.8*ω*_*o*_, *ξ* = 0.03, no perforation effect, single cavity).

	*κ* = 1	*κ* = 5	*κ* = 10	*κ* = 20
One mode	0.04780	0.04874	0.05010	0.05006
Four modes	0.04813	0.04908	0.05045	0.05038
Nine modes	0.04814	0.04909	0.05045	0.05039

**Table 2:** Absorption coefficient convergence for various numbers of modes used (*ω* = *ω*_*o*_, *ξ* = 0.03, no perforation effect, single cavity).

	*κ* = 1	*κ* = 5	*κ* = 10	*κ* = 20
One mode	0.92461	0.68454	0.47455	0.29215
Four modes	0.93382	0.69734	0.48392	0.29714
Nine modes	0.93401	0.69763	0.48413	0.29725

**Table 3:** Absorption coefficient convergence for various numbers of modes used (*ω* = 2*ω*_*o*_, *ξ* = 0.03, no perforation effect, single cavity).

	*κ* = 1	*κ* = 5	*κ* = 10	*κ* = 20
One mode	0.00523	0.00540	0.00576	0.00648
Four modes	0.00520	0.00537	0.00572	0.00644
Nine modes	0.00520	0.00537	0.00572	0.00644

**Table 4:** Absorption coefficient convergence for various level solutions (*ω* = 0.8*ω*_*o*_, *ξ* = 0.03, no perforation effect, single cavity).

	*κ* = 1	*κ* = 5	*κ* = 10	*κ* = 20
Zero level solution	0.04814	0.04914	0.05067	0.05123
1^st^ level solution	0.04813	0.04908	0.05045	0.05038
2^nd^ level solution	0.04813	0.04908	0.05045	0.05038

**Table 5:** Absorption coefficient convergence for various level solutions (*ω* = *ω*_*o*_, *ξ* = 0.03, no perforation effect, single cavity).

	*κ* = 1	*κ* = 5	*κ* = 10	*κ* = 20
Zero level solution	0.93381	0.70037	0.30973	0.30973
1^st^ level solution	0.93382	0.69734	0.48392	0.29714
2^nd^ level solution	0.93382	0.69734	0.48394	0.29724

**Table 6:** Absorption coefficient convergence for various level solutions (*ω* = 2*ω*_*o*_, *ξ* = 0.03, no perforation effect, single cavity).

	*κ* = 1	*κ* = 5	*κ* = 10	*κ* = 20
Zero level solution	0.00520	0.00537	0.00572	0.00644
1^st^ level solution	0.00520	0.00537	0.00572	0.00644
2^nd^ level solution	0.00520	0.00537	0.00572	0.00644

**Fig 5:**
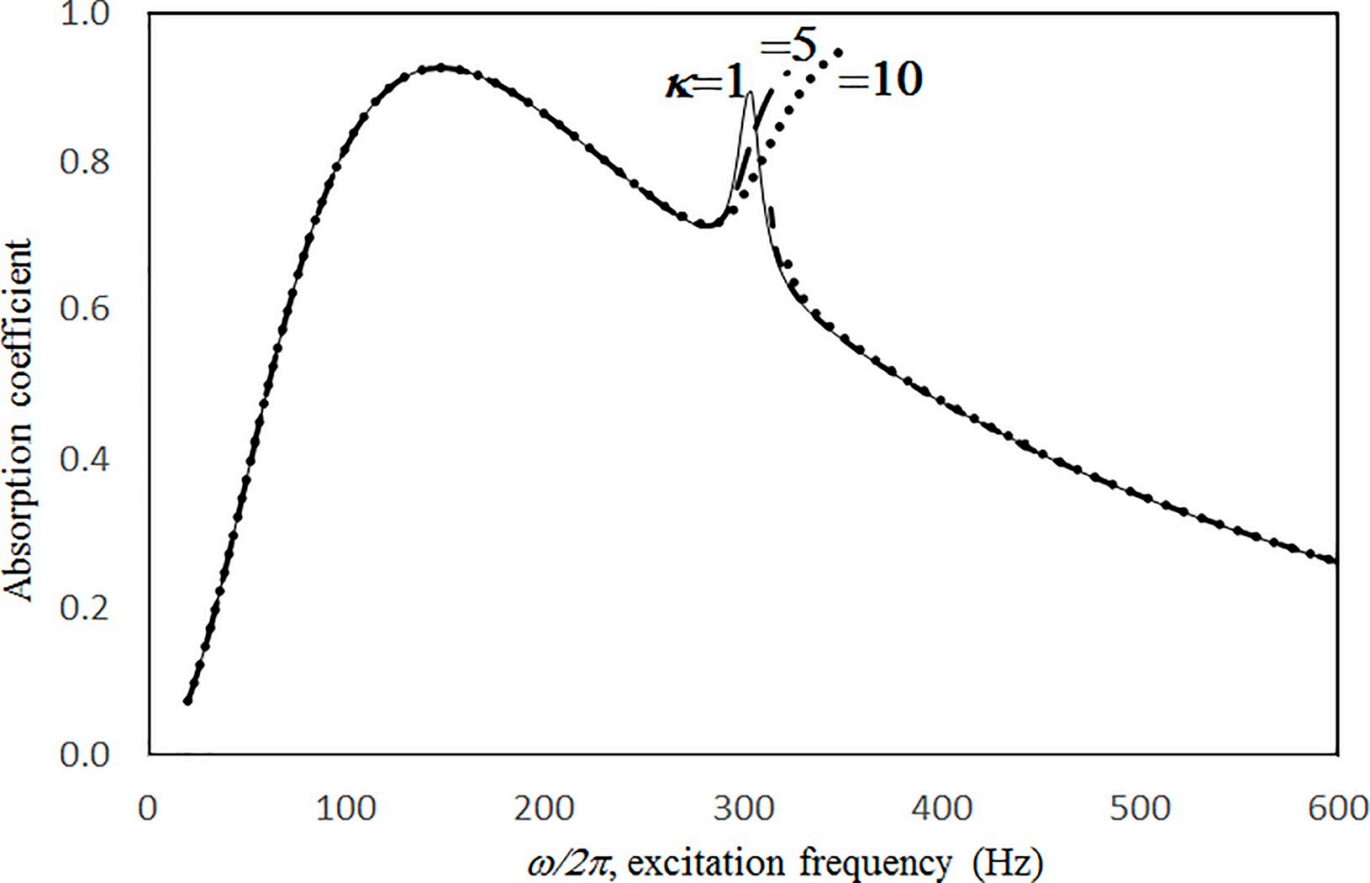
Absorption coefficient versus excitation frequency for various excitation magnitudes (*τ* = 2.5mm, *a = b = c =* 0.2m, *ξ* = 0.01, *σ =* 1%, *δ* = 0.5mm, single cavity, linear perforation and large amplitude vibration).

**Fig 6:**
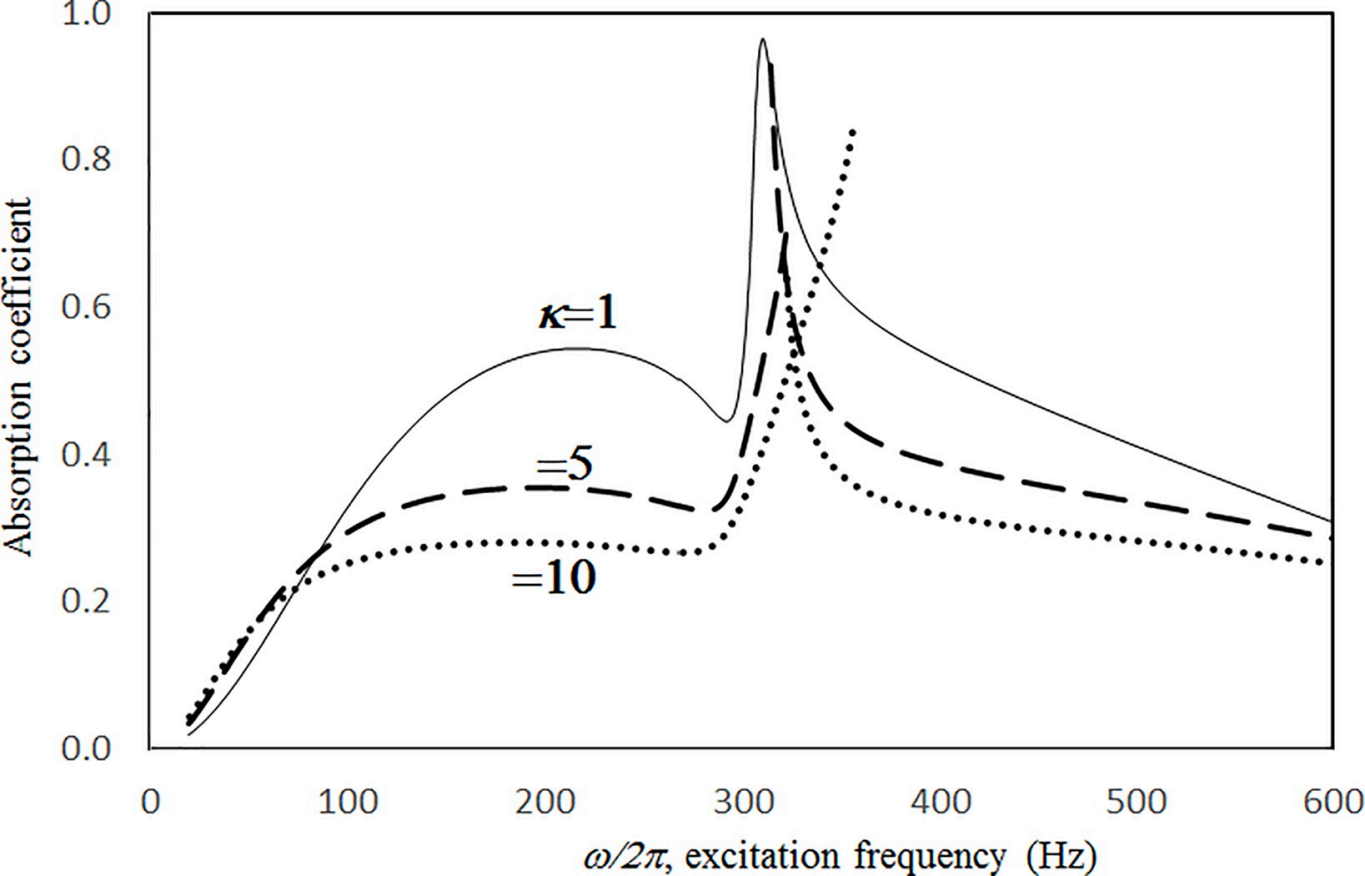
Absorption coefficient versus excitation frequency for various excitation magnitudes (*τ* = 2.5mm, *a = b = c =* 0.2m, *ξ* = 0.01, *σ =* 1%, *δ* = 0.5mm, three cavities of equal depth, nonlinear perforation and large amplitude vibration).

**Fig 7:**
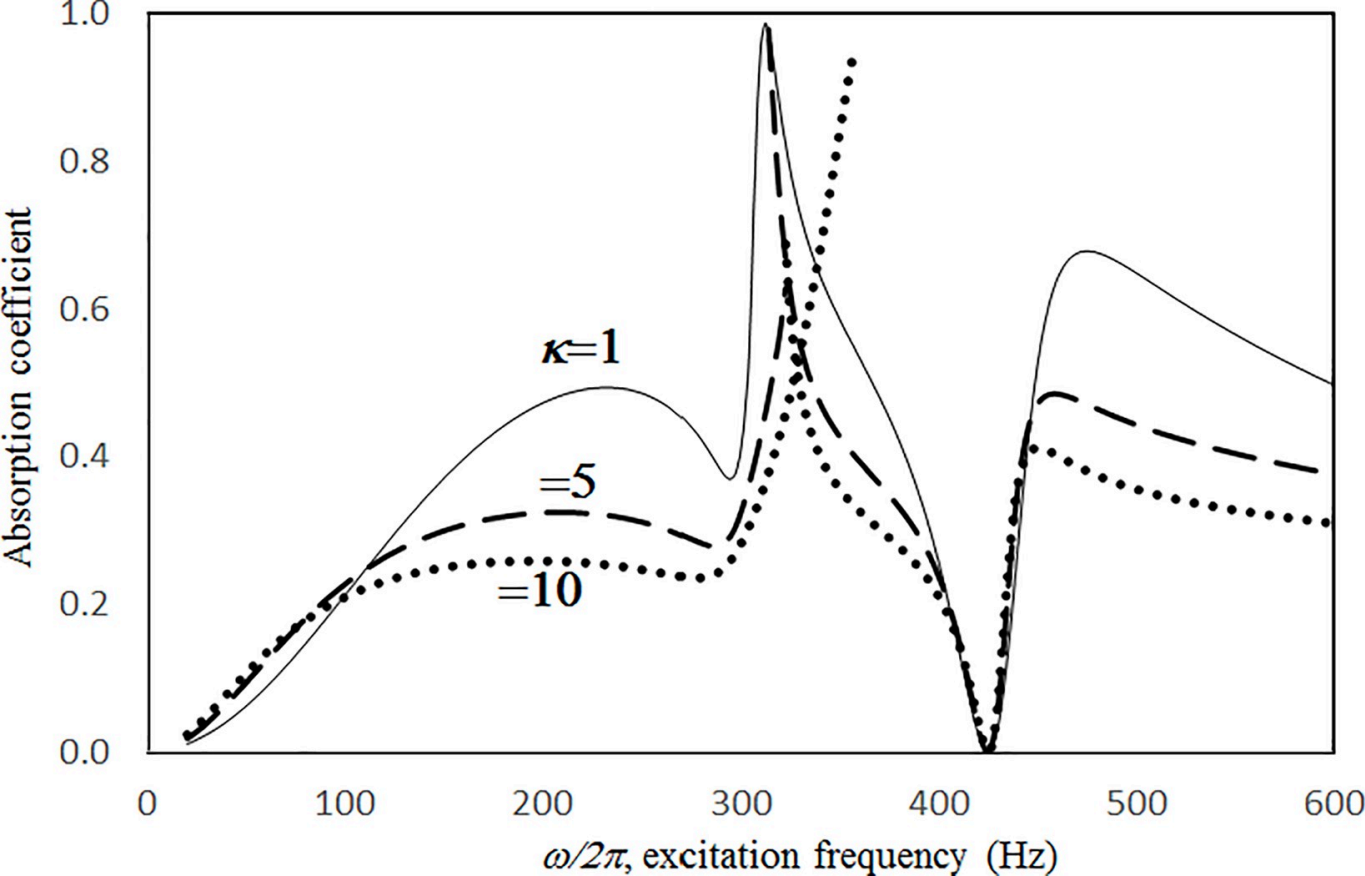
Absorption coefficient versus excitation frequency for various excitation magnitudes (*τ* = 2.5mm, *a = b =* 0.2m, *c*_*1*_ *= c*_*3*_ *=* 0.1m, *c*_*2*_ *=* 0.4m, *ξ* = 0.01, *σ =* 1%, *δ* = 0.5mm, three cavities of unequal depths, nonlinear perforation and large amplitude vibration).

**Fig 8:**
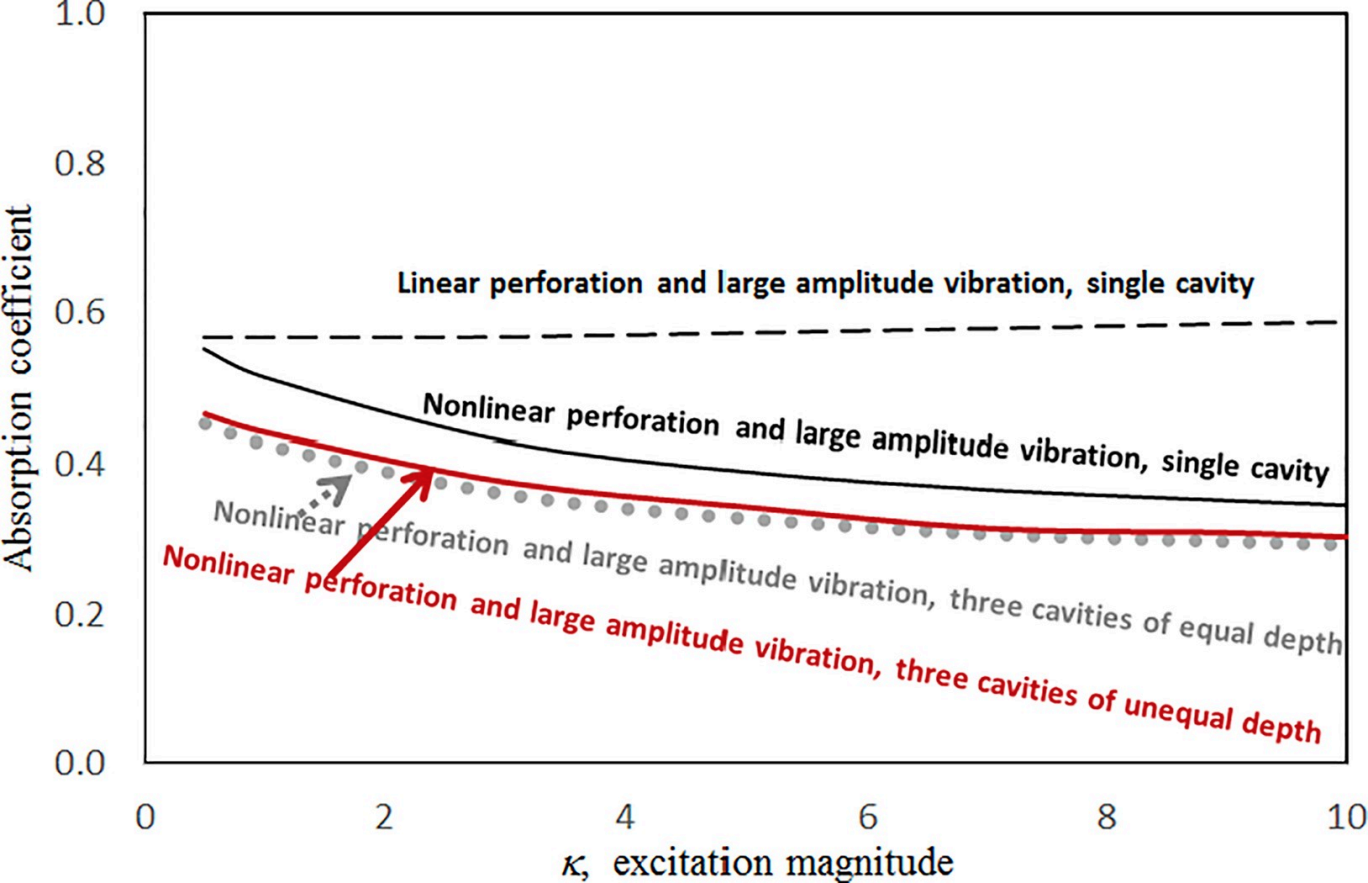
Average absorption coefficient versus excitation magnitude for various nonlinear cases (*τ* = 2.5mm, *a = b = c =* 0.2m, *ξ* = 0.01, *σ =* 1%, *δ* = 0.5mm).

Figs 4 through 7 show the absorption coefficients plotted against the excitation frequency for various excitation magnitudes. The four cases include 1) nonlinear perforation, large-amplitude vibration, and a single cavity; 2) linear perforation, large-amplitude vibration, and a single cavity; 3) nonlinear perforation, large-amplitude vibration, and three cavities of equal depth; and 4) nonlinear perforation, large-amplitude vibration, and three cavities of unequal depth. The damping ratio *ξ* is 0.01. The panel thickness is 2.5 mm. The hole diameter is 0.5 mm, and perforation ratio is 1%. In Fig 4, the absorption decreases along with the excitation magnitude. The absorption peaks around 300 Hz are caused by the panel resonance. It is noted that the well-known “jump phenomenon,” which is sometimes observed in various nonlinear oscillation problems, is also found in this nonlinear structural-acoustic study. In the cases of *κ =* 5 and 20, except around the resonant frequency, the absorption coefficient remains nearly unchanged from 100 to 600 Hz. In the case of *κ =* 1, except around the resonant frequency, the absorption decreases from 100 to 600 Hz. In Fig 5, the absorption due to the linear perforation peaks around 130 Hz and then decreases monotonically. Another absorption peak appears at around 300 Hz due to the large-amplitude vibration. The excitation magnitude affects only the peak absorption around 300 Hz. The nonlinear peak due to panel vibration can be seen in the case of *κ =* 20. In other frequency ranges, the three curves of various excitation magnitudes are almost identical. A comparison of the curves in Figs 4 and 6 shows that in the case of three cavities with equal depth, the three absorption curves at the off resonant range are slightly higher when the excitation frequency is higher than the corresponding peak frequency. Another absorption peak is observed around 210 Hz in the case of *κ =* 1. The comparison between the curves in Figs 6 and 7 shows that in the case of three cavities with unequal depths, an absorption trough appears around 420 Hz, and an absorption peak appears around 485 Hz. The absorption peak around 485 Hz is larger than that around 300 Hz, and both are due to the perforation effect. Fig 8 shows the average absorption (from 20 to 600 Hz) plotted against the excitation magnitude for these four cases. In the case of linear perforation, large-amplitude vibration, and a single cavity, the average absorption remains nearly unchanged as the excitation magnitude increases. In the other three cases, the absorption curves are very close and decrease monotonically. From *κ =* 0.5 to 2, the slopes of the absorption curves are deeper than those at the higher excitation magnitudes (i.e. *κ >* 2). The two absorption curves of three cavities are nearly identical and are always lower than the other two curves. From this result, it is known that the average absorption performance can be degraded by increasing the excitation magnitude to trigger a greater nonlinear perforation effect.

**Fig 9:**
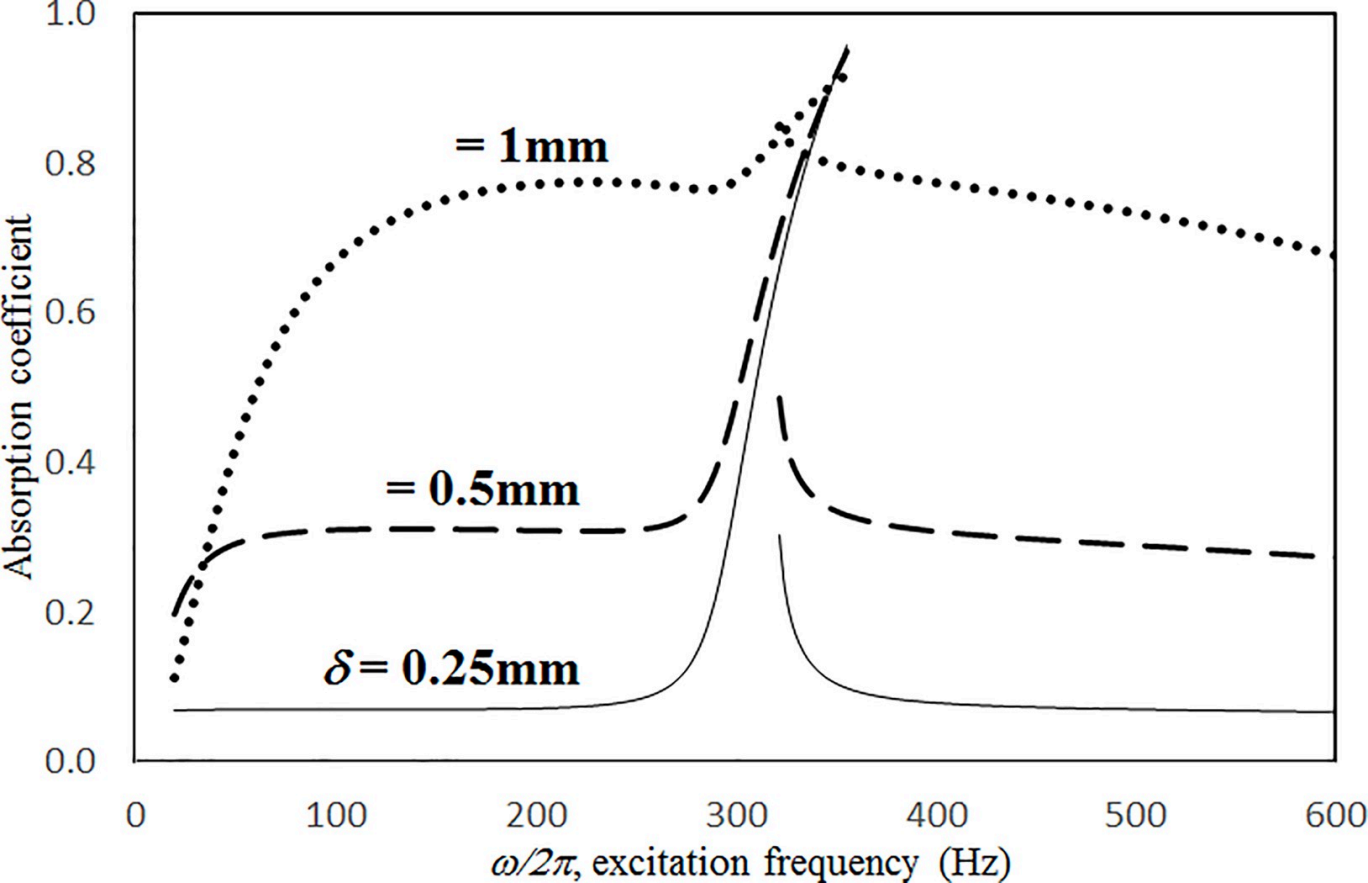
Absorption coefficient versus excitation frequency for various hole diameters (*τ* = 2.5mm, *a = b = c =* 0.2m, *ξ* = 0.01, *κ* = 10, single cavity, nonlinear perforation and large amplitude vibration).

**Fig 10:**
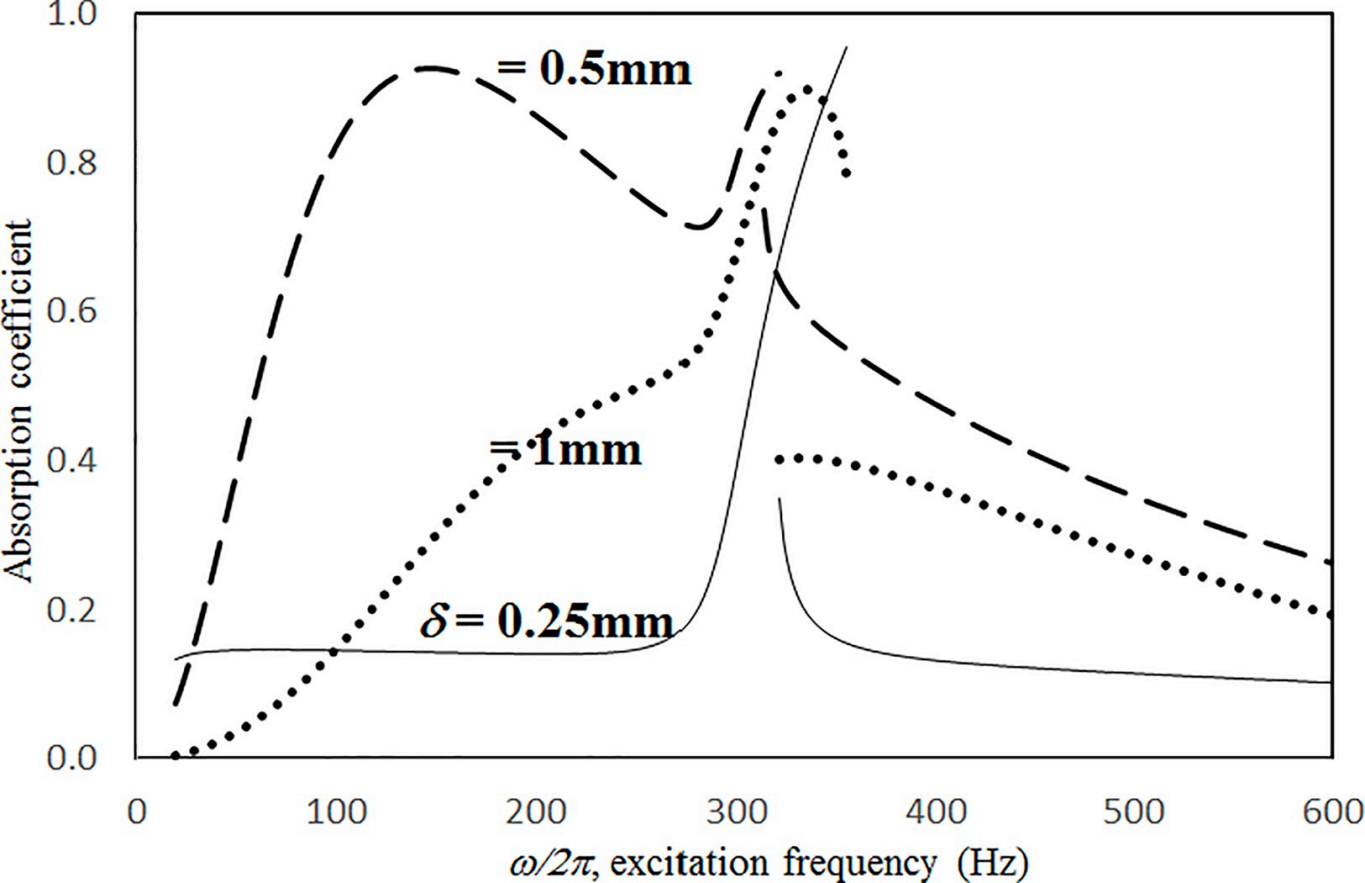
Absorption coefficient versus excitation frequency for various hole diameters (*τ* = 2.5mm, *a = b = c =* 0.2m, *ξ* = 0.01, *κ* = 10, single cavity, linear perforation and large amplitude vibration).

**Fig 11:**
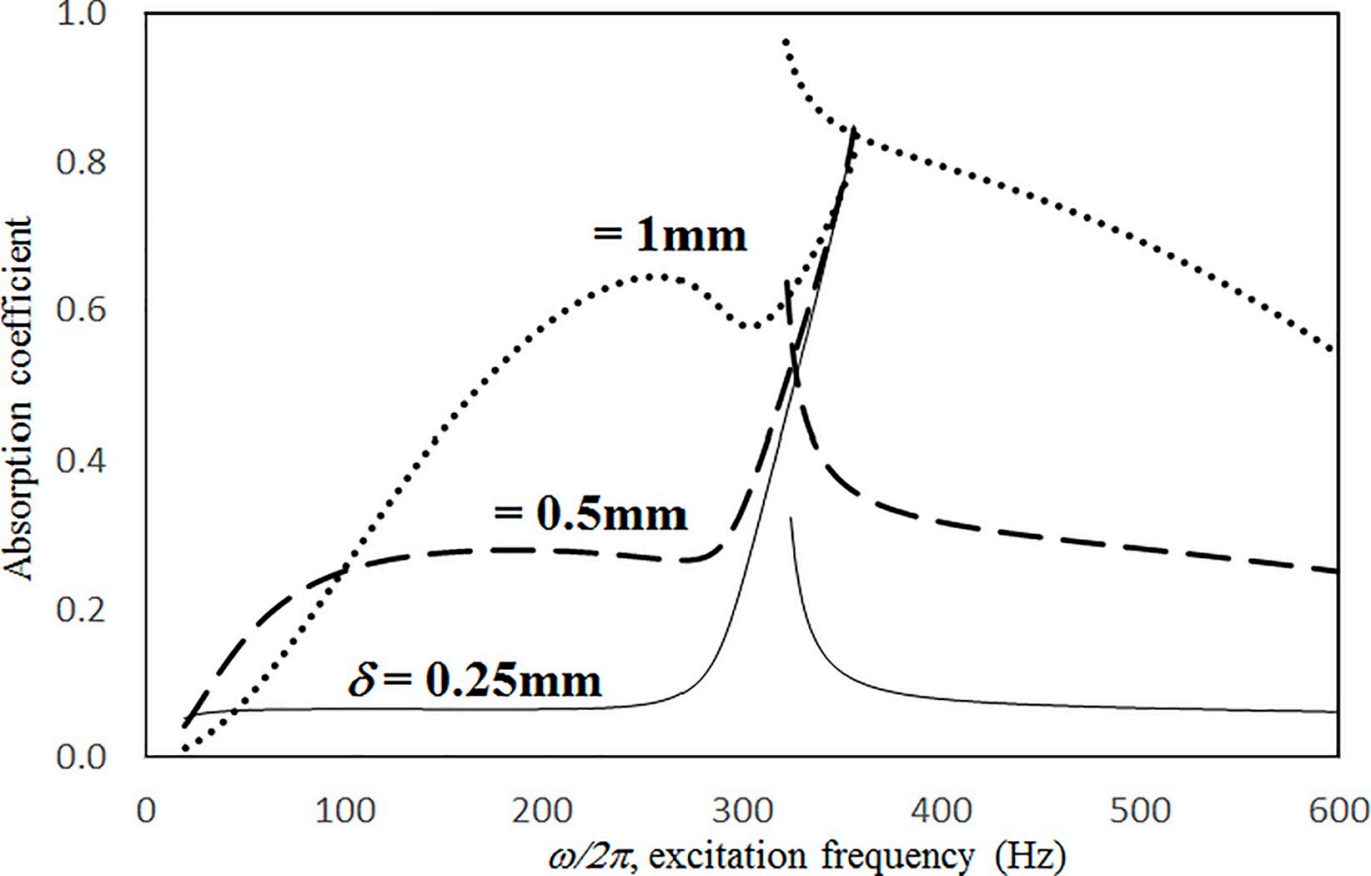
Absorption coefficient versus excitation frequency for various hole diameters (*τ* = 2.5mm, *a = b = c =* 0.2m, *ξ* = 0.01, *κ* = 10, three cavities of equal depth, nonlinear perforation and large amplitude vibration).

**Fig 12:**
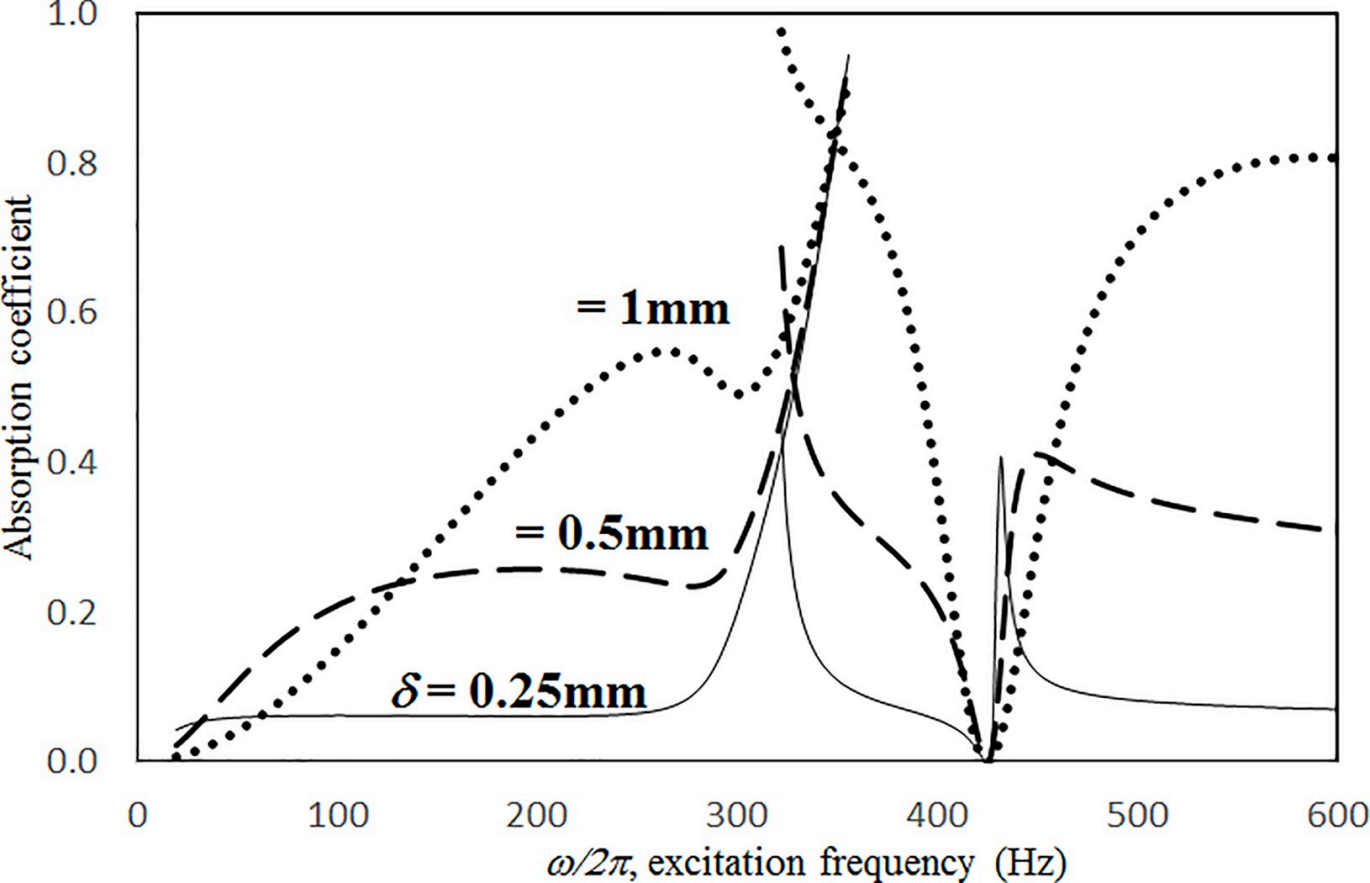
Absorption coefficient versus excitation frequency for various hole diameters (*τ* = 2.5mm, *a = b =* 0.2m, *c*_*1*_ *= c*_*3*_ *=* 0.1m, *c*_*2*_ *=* 0.4m, *ξ* = 0.01, *κ* = 10, three cavities of unequal depths, nonlinear perforation and large amplitude vibration).

**Fig 13:**
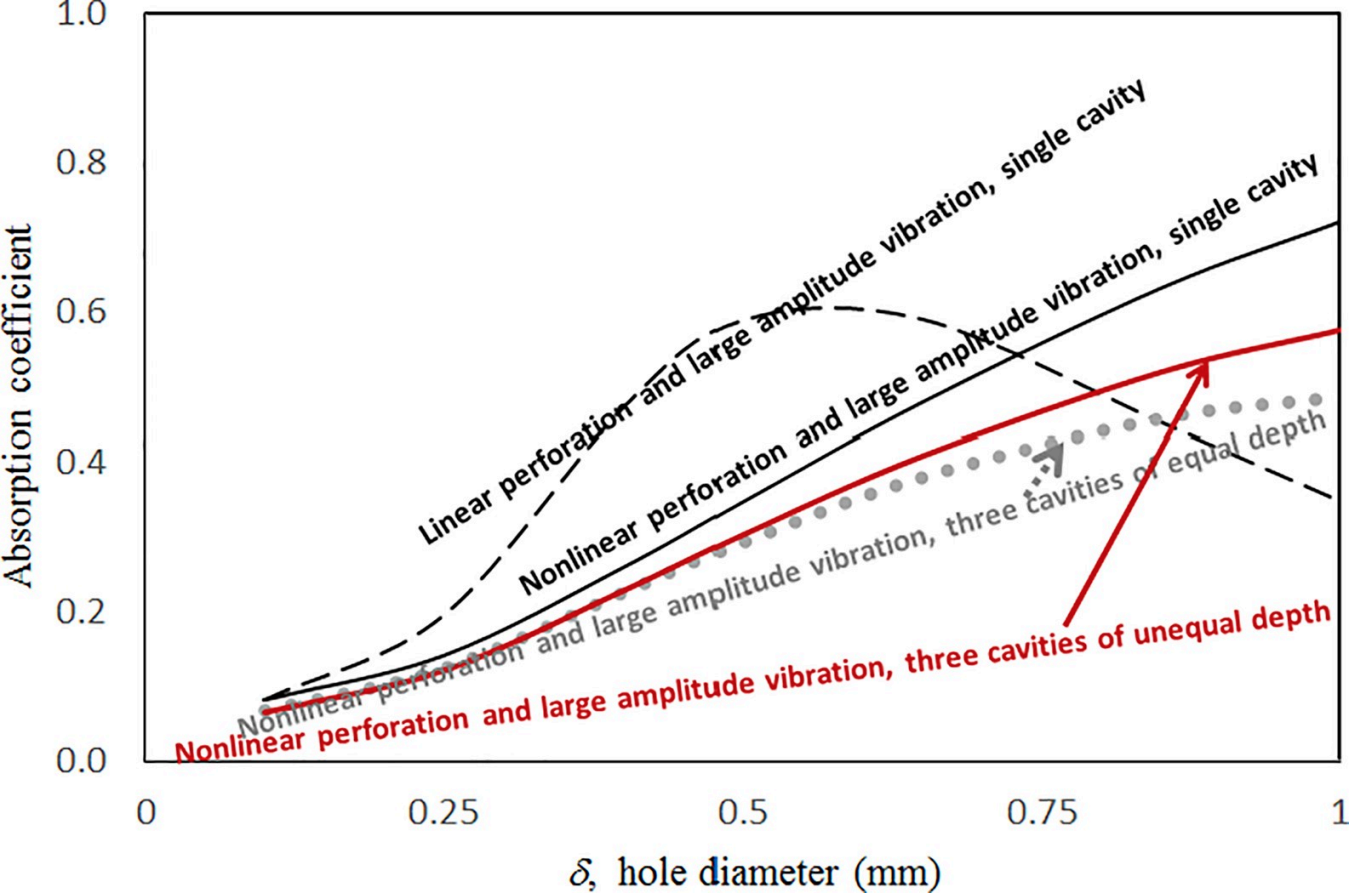
Average absorption coefficient versus hole diameter for various nonlinear cases (*τ* = 2.5mm, *a = b = c =* 0.2m, *ξ* = 0.01, *κ* = 10).

Figs 9 through 12 show the absorption coefficients plotted against the excitation frequency for various hole diameters. The four cases include 1) nonlinear perforation, large-amplitude vibration, and a single cavity; 2) linear perforation, large-amplitude vibration, and a single cavity; 3) nonlinear perforation, large-amplitude vibration, and three cavities of equal depth; and 4) nonlinear perforation, large-amplitude vibration, and three cavities of unequal depth. The dimensionless excitation parameter *κ* is 10. The panel thickness is 2.5 mm. In Fig 9, the larger the diameter of the hole, the greater the nonlinear perforation and absorption. Except around the resonant frequency, the absorption coefficient remains nearly unchanged from 100 to 600 Hz for hole diameters of 0.25 and 0.5 mm. In addition, the absorption peak due to panel vibration is not significantly affected by the hole diameter, which is one of the key factors that controls the nonlinear perforation effect. A comparison of Figs 9 and 10 shows that the effects of hole diameter on linear and nonlinear perforation are completely different. In Fig 9, the absorption curves are shifted up by increasing the hole diameter. In Fig 10, the absorption peak due to linear perforation is induced around 140 Hz by increasing the hole diameter, but no such absorption peak is found in Fig 9. In Fig 10, the absorption decreases with the hole diameter within the frequency range from 350 to 600 Hz, whereas it remains almost constant in Fig 9. In addition, in Fig 10, the absorption peak value and peak frequency due to panel vibration are slightly lowered by increasing the hole diameter, whereas they are nearly unchanged in Fig 9. A comparison between the curves in Figs 9 and 11 shows that in the case of three cavities with equal depth, the “jump up phenomenon” is observed from low to high frequencies (around 350 Hz) for a hole diameter of 1 mm, whereas the “jump down phenomenon” is found in the case of a single cavity. A small and wide absorption peak due to nonlinear perforation is found around 250 Hz in Fig 11, but no such peak is found in Fig 9. In Fig 11, the absorption changes substantially with hole diameters of 0.5 to 1 mm; the “jump down phenomenon” becomes the “jump up phenomenon” around 350 Hz; and the peak value becomes much higher. Note that the absorption curves for hole diameters of 0.25 and 0.5 mm in Figs 9 and 11 have similar appearances. The absorption curves in Figs 11 and 12 are very similar except around 420 Hz, where a trough is induced by the cavities of unequal depth. Fig 13 shows the average absorption (from 20 to 600 Hz) plotted against the hole diameter for these four cases. In the case of linear perforation, large-amplitude vibration, and a single cavity, the average absorption curve differs greatly from the other three curves; its peak is observed around a hole diameter of 0.55 mm, whereas the other three absorptions increase monotonically over the whole range. Among the three absorption curves, the case of a single cavity is always the highest, and the case of three cavities with unequal depth is always the lowest (because of the absorption trough around 420 Hz in Fig 12). This result shows that the average absorption performance may be degraded by the three cavities of unequal depth.

**Fig 14:**
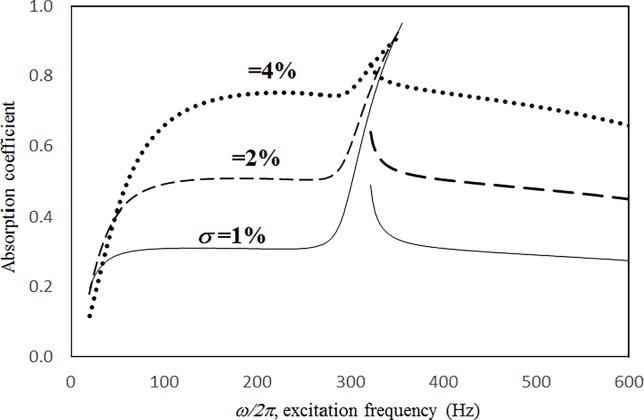
Absorption coefficient versus excitation frequency for various perforation ratios (*τ* = 2.5mm, *a = b = c =* 0.2m, *ξ* = 0.01, *κ* = 10, *δ* = 0.5mm, single cavity, nonlinear perforation and large amplitude vibration).

**Fig 15:**
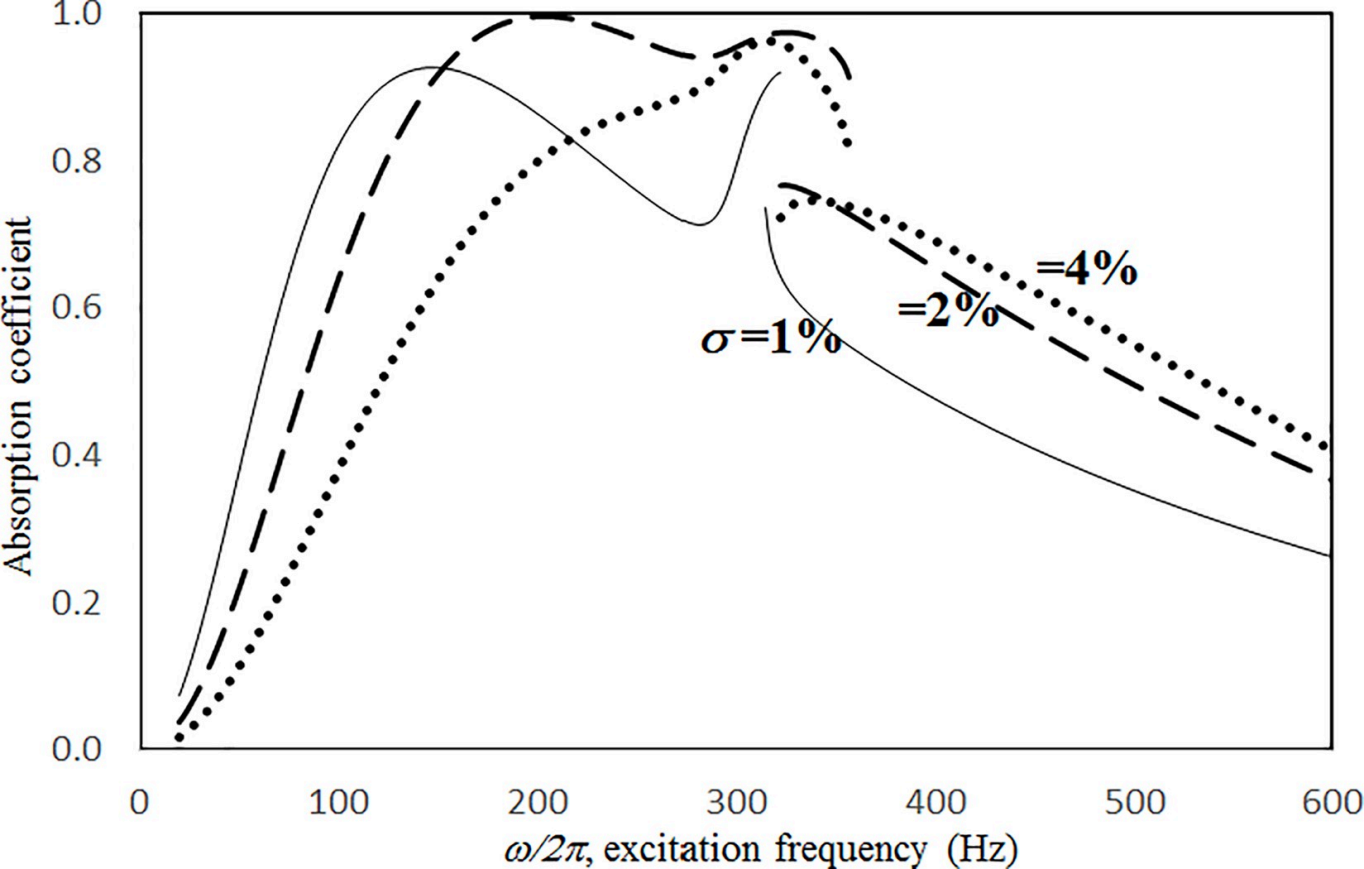
Absorption coefficient versus excitation frequency for various perforation ratios (*τ* = 2.5mm, *a = b = c =* 0.2m, *ξ* = 0.01, *κ* = 10, *δ* = 0.5mm, single cavity, linear perforation and large amplitude vibration).

**Fig 16:**
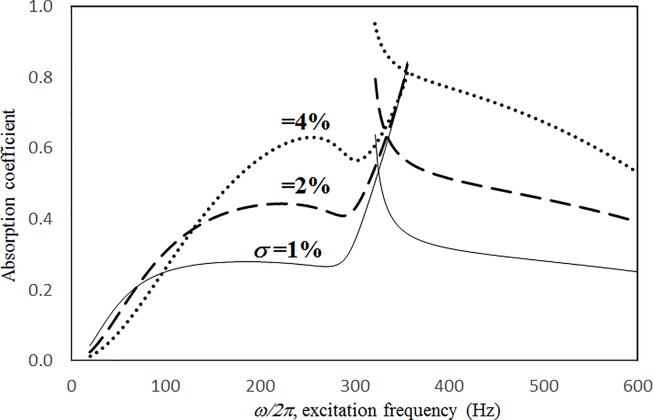
Absorption coefficient versus excitation frequency for various perforation ratios (*τ* = 2.5mm, *a = b = c =* 0.2m, *ξ* = 0.01, *κ* = 10, *δ* = 0.5mm, three cavities of equal depth, nonlinear perforation and large amplitude vibration).

**Fig 17:**
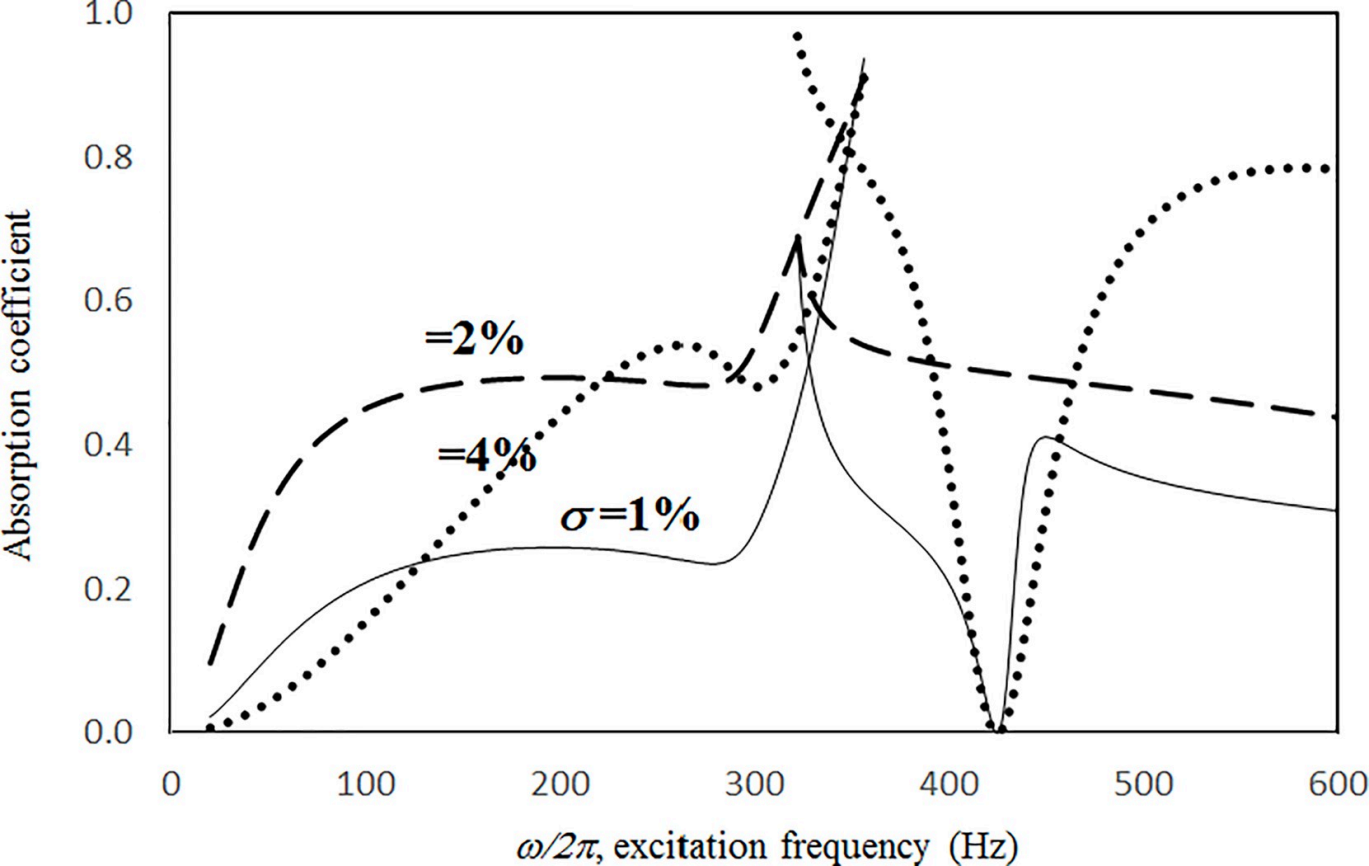
Absorption coefficient versus excitation frequency for various perforation ratios (*τ* = 2.5mm, *a = b =* 0.2m, *c*_*1*_ *= c*_*3*_ *=* 0.1m, *c*_*2*_ *=* 0.4m, *ξ* = 0.01, *κ* = 10, *δ* = 0.5mm, three cavities of unequal depths, nonlinear perforation and large amplitude vibration).

**Fig 18:**
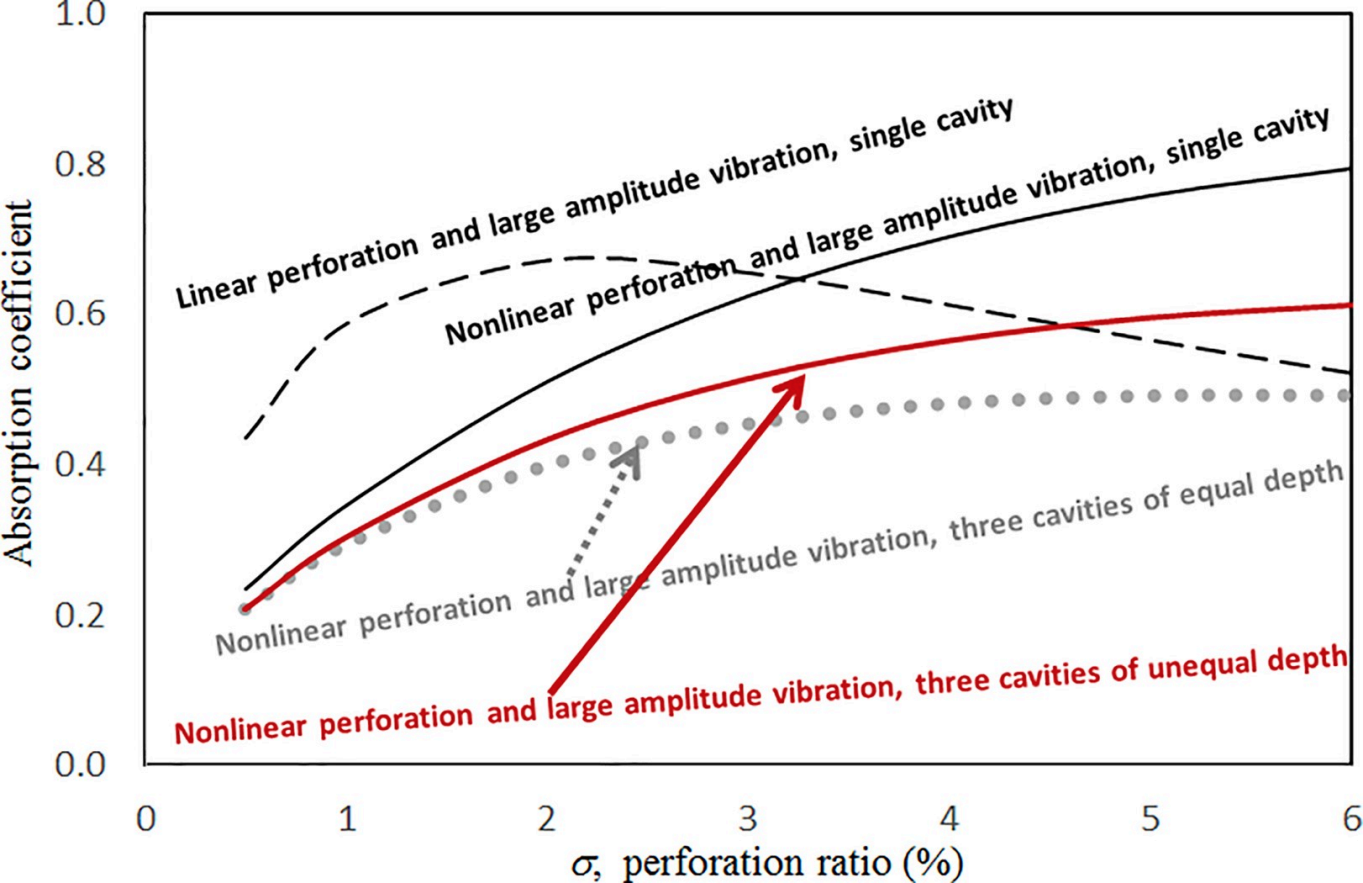
Average absorption coefficient versus perforation ratio for various nonlinear cases (*τ* = 2.5mm, *a = b = c =* 0.2m, *ξ* = 0.01, *κ* = 10, *δ* = 0.5mm).

Figs 14 through 17 show the absorption coefficients plotted against the excitation frequency for various perforation ratios. The four cases include 1) nonlinear perforation, large-amplitude vibration, and a single cavity; 2) linear perforation, large-amplitude vibration, and a single cavity; 3) nonlinear perforation, large-amplitude vibration, and three cavities of equal depth; and 4) nonlinear perforation, large-amplitude vibration, and three cavities of unequal depth. The dimensionless excitation parameter *κ* is 10. The panel thickness is 2.5 mm. In Fig 14, the higher the perforation ratio, the greater the nonlinear perforation and absorption. Except around the resonant frequency, the absorption coefficient remains nearly unchanged from 100 to 600 Hz for perforation ratios of 1% and 2%. In addition, the absorption peak due to panel vibration is not significantly affected by the perforation ratio, which is also one of the key factors that controls the nonlinear perforation effect. A comparison of Figs 14 and 15 shows that the effects of perforation ratio on linear and nonlinear perforation are completely different. In Fig 14, the absorption curves are shifted up by increasing the perforation ratio. In Fig 15, the absorption peaks due to linear perforation are induced around 140 and 190 Hz for perforation ratios of 2% and 4%, respectively, whereas no such absorption peak is found in Fig 14. In Fig 15, the absorption decreases along with the perforation ratio for the frequency range from 320 to 600 Hz but remains almost constant in Fig 14. In addition, in Fig 15, when the perforation ratio is 2%, the absorption peak bandwidth is optimized and the widest because the peaks due to panel vibration and perforation are close and coupled with each other. When the perforation ratio is 4%, the two peaks are so close that they look like a single peak, and the bandwidth is narrow. A comparison of the curves in Figs 14 and 16 shows that in the case of three cavities with equal depth, the “jump up phenomenon” is observed from low to high frequencies (around 350 Hz) for the perforation ratio of 4%, whereas the “jump down phenomenon” is found in the case of a single cavity. A small and wide absorption peak due to nonlinear perforation can be found around 250 Hz in Fig 16, but no such peak is found in Fig 14. As in Fig 11, the absorption in Fig 16 is substantially changed from perforation ratios of 2% to 4%; the “jump down phenomenon” becomes the “jump up phenomenon” around 350 Hz; and the peak value increases significantly. The absorption curves of the perforation ratios of 1% and 2% in Figs 14 and 16 also appear very similar. The absorption curves in Figs 16 and 17 are very similar except around 420 Hz, where a trough is induced by the cavities of unequal depth. Fig 18 shows the average absorption (from 20 to 600 Hz) plotted against the perforation ratio for these four cases. In the case of linear perforation, large-amplitude vibration, and a single cavity, the average absorption curve differs greatly from the other three curves; its peak is observed around a perforation ratio of 2%, whereas the other three absorptions increase monotonically over the whole range. Among the three absorption curves of various perforation ratios, the one with a single cavity is always the highest, whereas the one with three cavities of unequal depth is always the lowest (it is also caused by the absorption trough in Fig 17).

**Fig 19:**
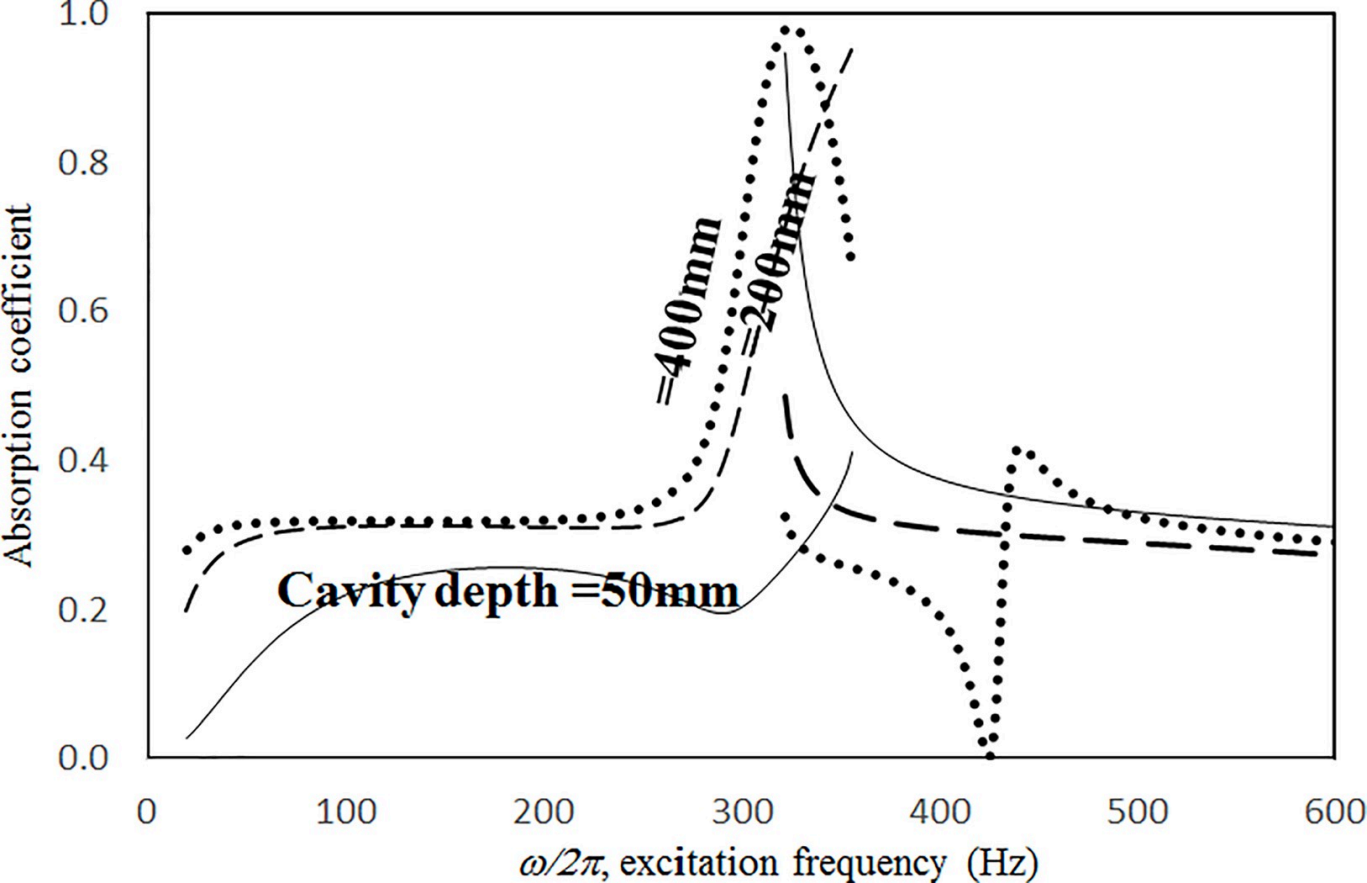
Absorption coefficient versus excitation frequency for various cavity depths (*τ* = 2.5mm, *a = b =* 0.2m, *ξ* = 0.01, *κ* = 10, *δ* = 0.5mm, *σ =* 1%, single cavity, nonlinear perforation and large amplitude vibration).

**Fig 20:**
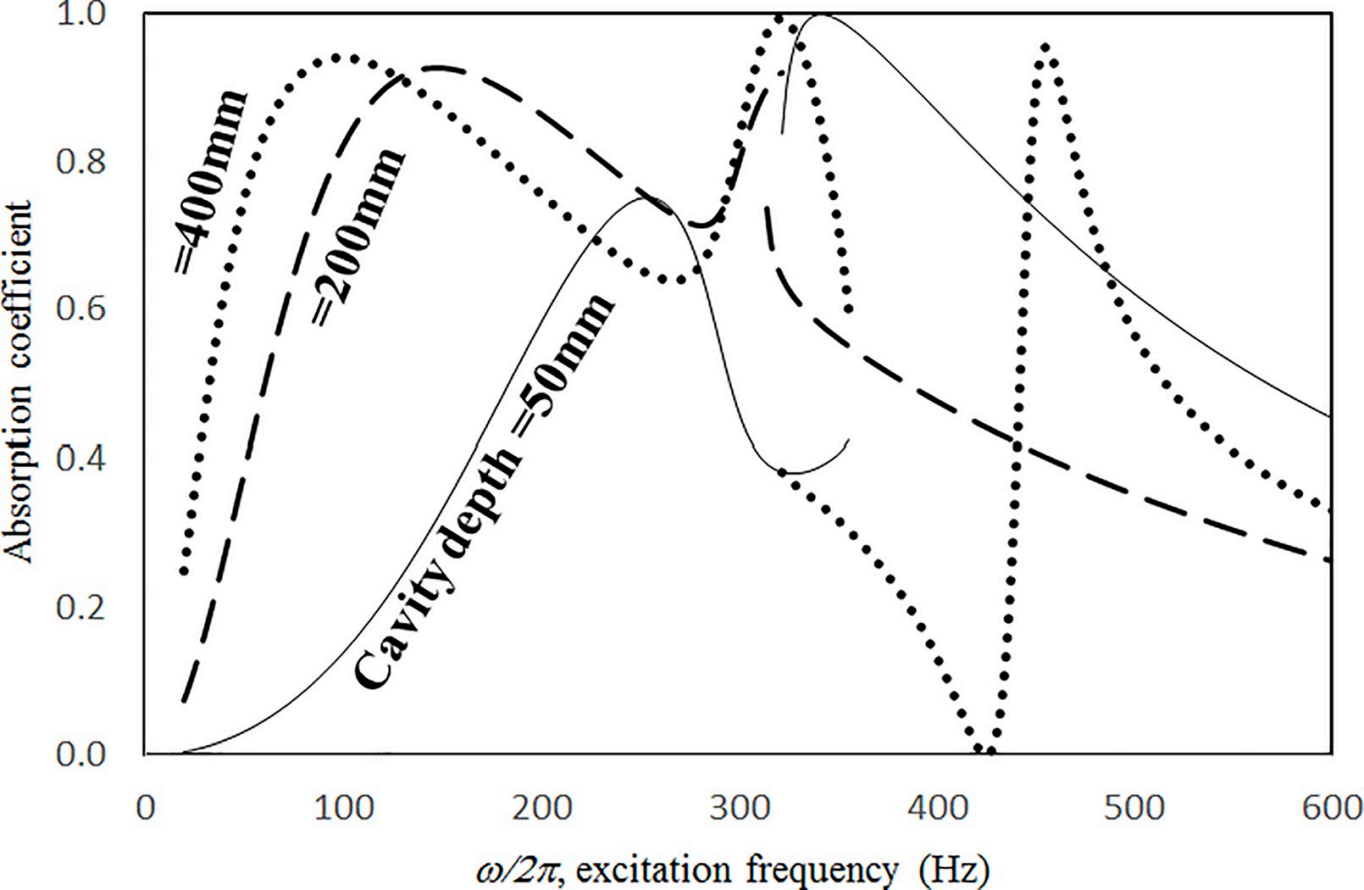
Absorption coefficient versus excitation frequency for various cavity depths (*τ* = 2.5mm, *a = b =* 0.2m, *ξ* = 0.01, *κ* = 10, *δ* = 0.5mm, *σ =* 1%, single cavity, linear perforation and large amplitude vibration).

**Fig 21:**
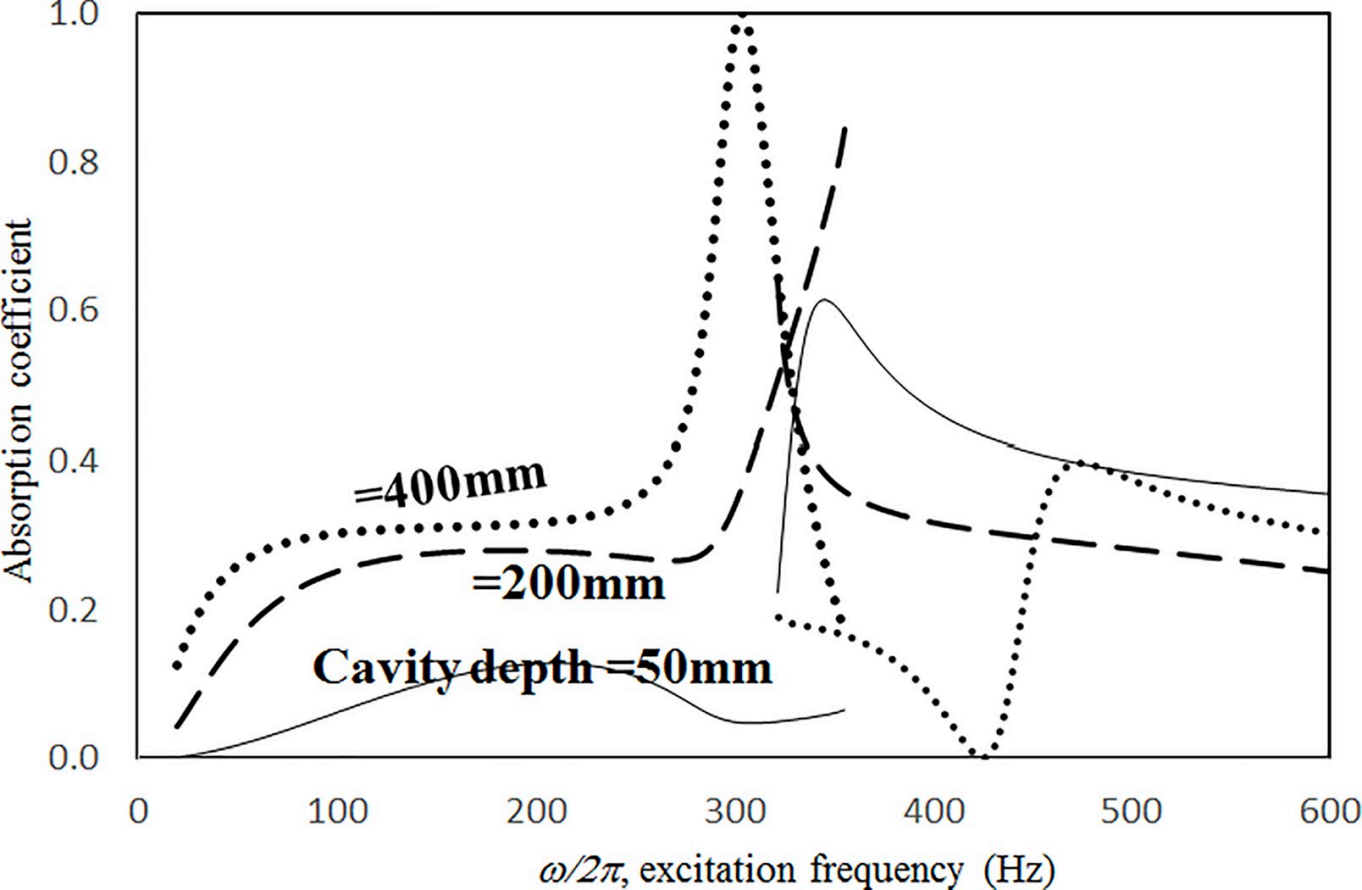
Absorption coefficient versus excitation frequency for various cavity depths (*τ* = 2.5mm, *a = b =* 0.2m, *ξ* = 0.01, *κ* = 10, *δ* = 0.5mm, *σ =* 1%, three cavities of equal depth, nonlinear perforation and large amplitude vibration).

**Fig 22:**
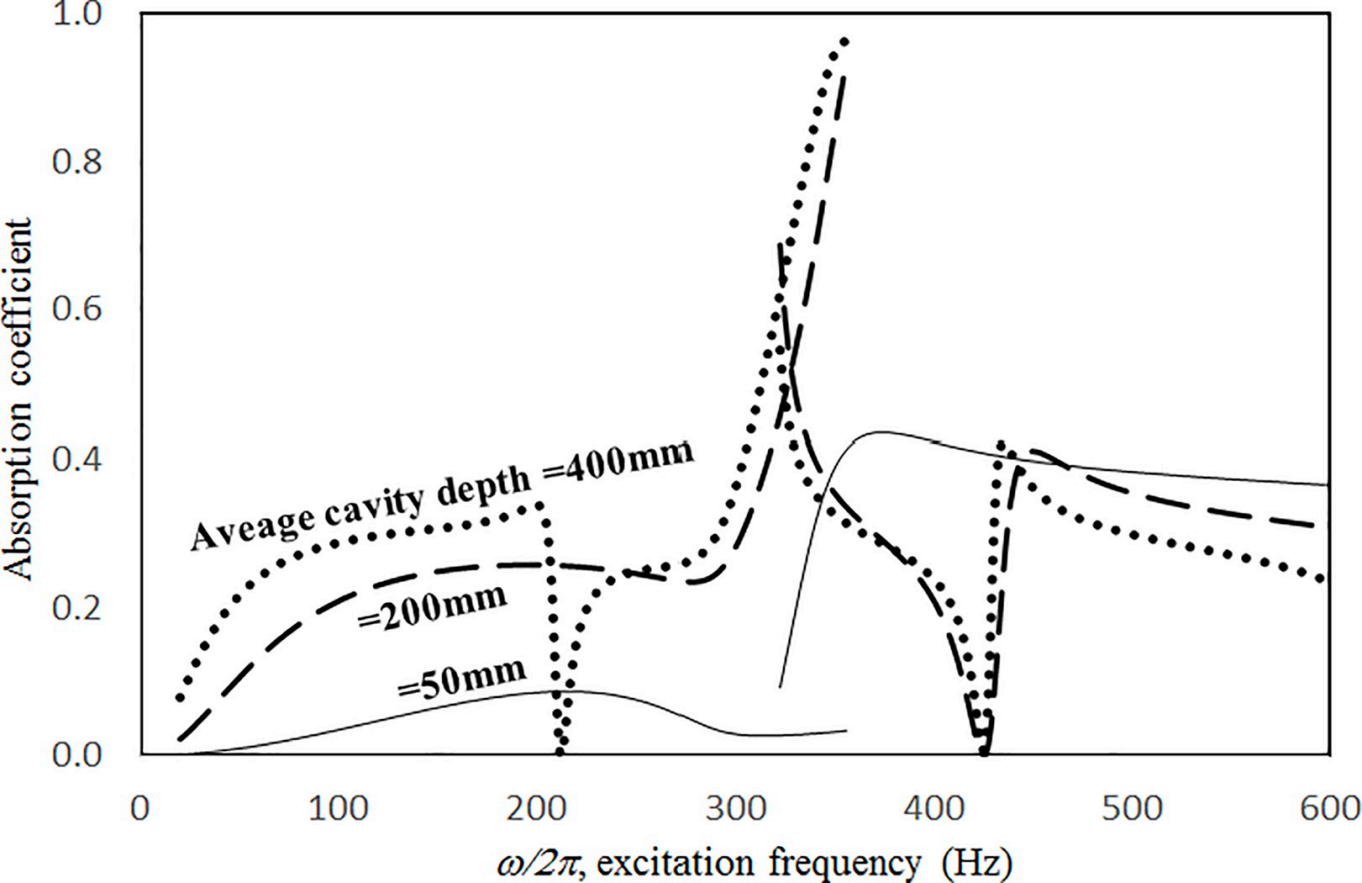
Absorption coefficient versus excitation frequency for various average cavity depths (*τ* = 2.5mm, *a = b =* 0.2m, *ξ* = 0.01, *κ* = 10, *δ* = 0.5mm, *σ =* 1%, three cavities of unequal depths, nonlinear perforation and large amplitude vibration).

**Fig 23:**
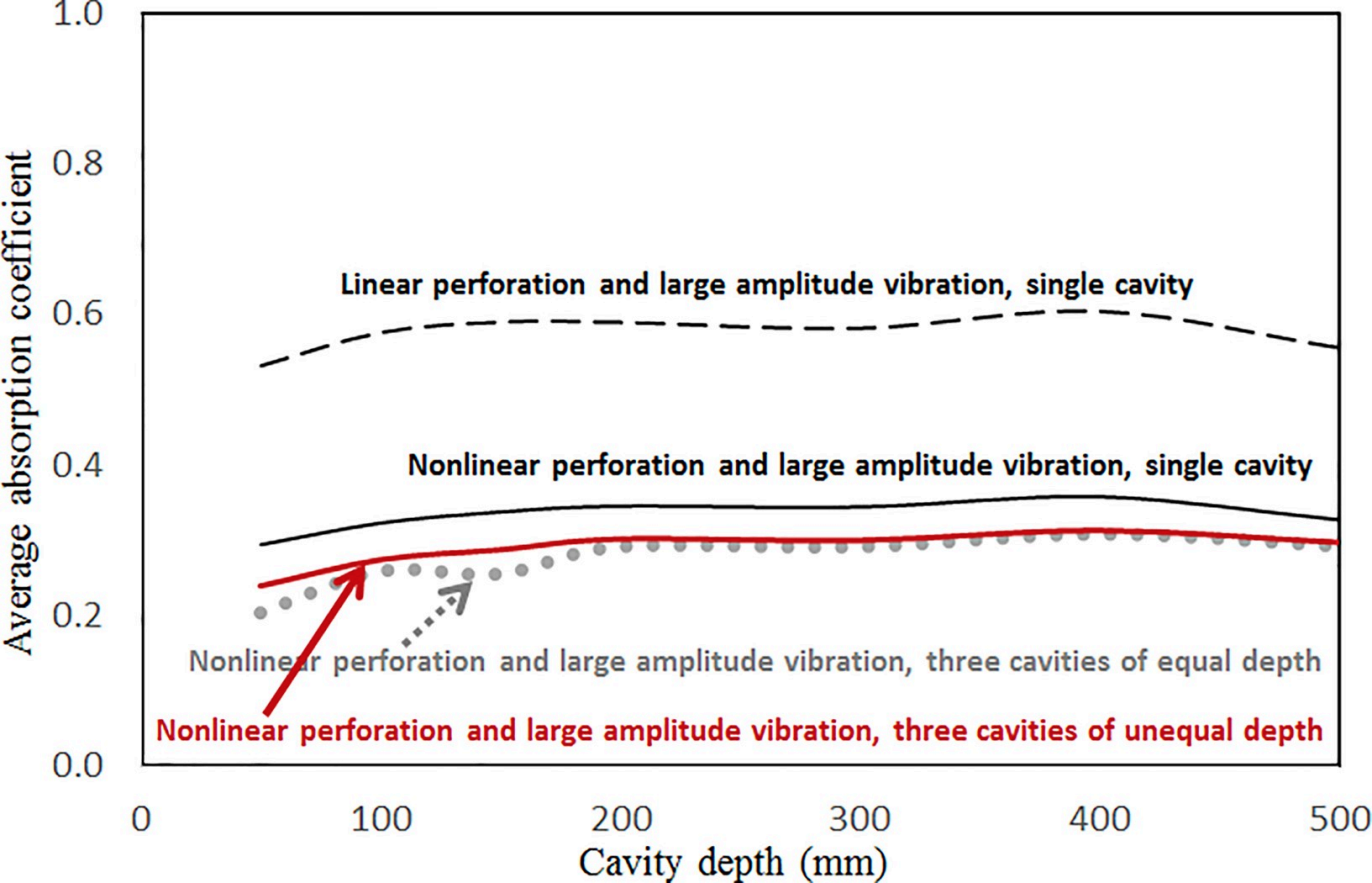
Average absorption coefficient versus cavity depth for various nonlinear cases (*τ* = 2.5mm, *a = b =* 0.2m, *ξ* = 0.01, *κ* = 10, *δ* = 0.5mm, *σ =* 1%,).

Figs 19 through 22 show the absorption coefficients plotted against the excitation frequency for various cavity depths. The four cases include 1) nonlinear perforation, large-amplitude vibration, and a single cavity; 2) linear perforation, large-amplitude vibration, and a single cavity; 3) nonlinear perforation, large-amplitude vibration, and three cavities of equal depth; and 4) nonlinear perforation, large-amplitude vibration, and three cavities of unequal depth. The dimensionless excitation parameter *κ* is 10. The panel thickness is 2.5 mm. The hole diameter is 0.5 mm, and the perforation ratio is 1%. In Fig 19, the panel resonant behavior is affected by changing the cavity depth. In the case of a cavity depth of 400 mm, the resonant frequency range is around 350 Hz, the peak appears quite linear, and a trough appears around 420 Hz. In the case of a cavity depth of 200 mm, the peak appears nonlinear, and the “jump down phenomenon” occurs. In the case of a cavity depth of 150 mm, the peak also appears nonlinear, but the “jump up phenomenon” occurs instead of the “jump down phenomenon.” Generally, the absorption coefficient remains nearly unchanged in the nonresonant frequency ranges. The comparison between Figs 19 and 20 shows that the effects of cavity depth on linear and nonlinear perforation are completely different. In Fig 20, the absorption peak due to perforation is directly affected by the cavity depth: the shallower the cavity, the higher the peak frequency. Note that the frequency of the peak due to panel vibration is not significantly affected by the cavity depth. Similar to the observations in Fig 19, the nonlinear behavior of the absorption peak due to panel vibration changes from the linear peak, “jump down phenomenon” and then “jump up phenomenon” by setting the cavity depth from 50 to 400 mm. A trough with a cavity depth of 400 mm is found on the absorption curve, which is similar to the one in Fig 19. Generally, the absorption curves in Fig 21 are quite similar to those in Fig 19. In Fig 22, the three cavity depths of the solid line are 25 mm, 100 mm, and 25 mm, respectively (i.e., average depth of 50 mm); the three cavity depths of the dashed line are 100 mm, 400 mm, and 100 mm, respectively (i.e. average depth of 200 mm); and the three cavity depths of the solid line are 200 mm, 800 mm, and 200 mm, respectively (i.e., average depth of 400 mm). In Fig 22, the solid curve is similar to the one with a cavity depth of 50 mm in Fig 21, but the absorption peak due to panel vibration is lower. The dashed curve is similar to the case of a cavity depth of 200 mm in Fig 21, but a trough is found around 420 Hz that is caused by the shortest cavity depth. Generally, the dotted curve appears similar to the dashed curve except that one more trough appears around 200 Hz. Fig 23 shows the average absorption (from 20 to 600 Hz) plotted against the cavity depth for these four cases. In the case of linear perforation, large-amplitude vibration, and a single cavity, the average absorption is significantly higher than the other three curves, which are quite close to each other. Generally, the absorptions of various curves vary little within the frequency range concerned, and no peaks or troughs can be found on them. In other words, it is implied that the cavity depth would not significantly affect the average absorption.

## 4 Conclusions

This study investigated the sound absorption of a perforated panel absorber backed by a single cavity or multiple cavities. The structural/acoustic formulation was developed from partial differential equations, which represent the large-amplitude vibration of a panel absorber and pressure-dependent absorption. The results obtained from the proposed harmonic balance method and numeric integration method are generally consistent. The effects of the excitation magnitude, cavity depth, perforation ratio, and hole diameter on the sound absorption of a panel absorber were investigated. The results show that 1) perforation nonlinearity is a very important factor that affects the absorption of a panel absorber at the off structural resonant frequency range. The settings for the hole diameter, perforation ratio, and cavity depth for optimum absorption would differ greatly if perforation nonlinearity is present; 2) the “jump up phenomenon,” which does not occur in the case of linear perforation, is observed when perforation nonlinearity is considered; and 3) one or more absorption troughs, which would worsen the average absorption performance, may exist in the cases of multiple cavities. In conclusion, the effects of large-amplitude vibration and pressure-dependent absorption must be considered.

## References

[1] Maa D.Y.: Potential of microperforated panel absorber. *Journal of the Acoustical Society of America* 104(5), 2861–2866 (1998)

[2] Maa D.Y.: Theory and design of microperforated panel sound-absorbing constructions. *Scientia Sinica* 18(1), 55–71 (1975)

[3] Lee Y.Y., Lee E.W.M.: Widening the sound absorption bandwidths of flexible micro-perforated curved absorbers using structural and acoustic resonances. *International Journal of Mechanical Sciences* 49(8), 925–934 (2007)

[4] Lee Y.Y., Lee E.W.M., Ng C.F.: Sound absorption of a finite flexible micro-perforated panel backed by an air cavity, *Journal of Sound and Vibration* 287(1–2), 227–243 (2005)

[5] Kim H.S., Ma P.S. Kim S.R. Lee S.H., Seo Y.H.: A model for the sound absorption coefficient of multi-layered elastic micro-perforated plates. *Journal of Sound and Vibration* 430, 75–92 (2018)

[6] Maa D.Y.: Microperforated panel at high sound intensity *Proceedings of Internoise Yokohama*,*Japan*, 1511–1514 (1994)

[7] Lee Y,Y.: The effect of leakage on the sound absorption of a nonlinear perforated panel backed by a cavity. *International Journal of Mechanical Sciences* 107, 242–252 (2016)

[8] Kang J., Fuchs H.V.: Predicting the absorption of open weave textiles and micro-perforated membranes backed by an air space. *Journal of Sound and Vibration* 220(5), 905–920 (1999)

[9] Sakagami K., Matsutani K., Morimoto M.: Sound absorption of a double-leaf micro-perforated panel with an air-back cavity and a rigid-back wall: Detailed analysis with a Helmholtz-Kirchhoff integral formulation. *Applied Acoustics* 71(5), 411–417 (2010)

[10] Toyoda M., Takahashi D.: Reduction of acoustic radiation by impedance control with a perforated absorber system. *Journal of Sound and Vibration* 286(3), 601–614 (2005)

[11] Lee S.H., Ih J.G., Peat K.S.: A model of acoustic impedance of perforated plates with bias flow considering the interaction effect. *Journal of Sound and Vibration* 303(3–5), 741–752 (2007)

[12] Park S.H.: A design method of micro-perforated panel absorber at high sound pressure environment in launcher fairings. *Journal of Sound and Vibration* 332(3), 521–535 (2013)

[13] Chiang Y.K., Choy Y.S.: Acoustic behaviors of the microperforated panel absorber array in nonlinear regime under moderate acoustic pressure excitation. *Journal of the Acoustical Society of America* 143(1),538–549 (2018) 10.1121/1.5021334 29390793

[14] Wang F., Zhao L., Zhang Y.L., and Qiao Z.: Simplified aeroelastic model for fluid structure interaction between microcantilever sensors and fluid surroundings. *PLOS ONE* 10(4) Article Number: e0123860 (2015) 10.1371/journal.pone.0123860 25898213 PMC4405586

[15] Lee Y.Y., Ng C.F.: Sound insertion loss of stiffened enclosure plates using the finite element method and the classical approach. *Journal of Sound and Vibration* 217(2), 239–260 (1998).

[16] Lee Y.Y., Su R.K.L., Ng C.F., Hui C.K.: The effect of the modal energy transfer on the sound radiation and vibration of a curved panel: Theory and Experiment. *Journal of Sound and Vibration* 324, 1003–1015 (2009)

[17] Mao W.B., Caballero A., Mckay R., Primiano C., and Sun W.: Fully-coupled fluid-structure interaction simulation of the aortic and mitral valves in a realistic 3D left ventricle model. *PLOS ONE* 12(9), Article Number: e0184729 (2017) 10.1371/journal.pone.0184729 28886196 PMC5590990

[18] Bavo A.M., Rocatello G., Iannaccone F., Degroote J., Vierendeels J., and Segers P.: Fluid-structure interaction simulation of prosthetic aortic valves: comparison between immersed boundary and arbitrary lagrangian-eulerian techniques for the mesh representation. *PLOS ONE* 11(4), Article Number: e0154517 (2016) 10.1371/journal.pone.0154517 27128798 PMC4851392

[19] Yao L.Y., Yu D.J., Cui X.Y., and Zang X.G.: Numerical treatment of acoustic problems with the smoothed finite element method. *Applied Acoustics* 71(8), 743–753 (2010)

[20] Nehete D.V., Modak S.V., Gupta K.: Structural FE model updating of cavity systems incorporating vibro-acoustic coupling. *Mechanical Systems and Signal Processing*. 50–51, 362–379 (2015)

[21] Wang Y., Zhang J., Le V.: Vibroacoustic analysis of a rectangular enclosure bounded by a flexible panel with clamped boundary condition. *Shock and Vibration* Article Number: 872963 (2014)

[22] Pan J., Elliott S.J., Baek K.H.: Analysis of low frequency acoustic response in a damped rectangular enclosure. *Journal of Sound and Vibration* 223(4), 543–566 (1999)

[23] Cui X.Y., Hu X., Wang G., Li G.Y.: An accurate and efficient scheme for acoustic structure interaction problems based on unstructured mesh. *Computer Methods in Applied Mechanics and Engineering* 317, 1122–1145 (2017).

[24] Wang G., Cui X.Y., Liang Z.M., and Li G.Y.: A coupled smoothed finite element method (S-FEM) for structural-acoustic analysis of shells. *Engineering Analysis with Boundary Elements* 61, 207–217 (2015)

[25] Chen M.X., Zhang C., Tao X.F., Deng N.Q.: Structural and acoustic responses of a submerged stiffened conical shell. *Shock and Vibration* Article Number: 954253 (2014)

[26] Lee Y.Y.:Free vibration analysis of a nonlinear panel coupled with extended cavity using the multi-level residue harmonic balance method. *Thin-Walled Structures* 98, 332–336 (2016)

[27] Hui C.K., Lee Y.Y., Reddy J.N.: Approximate elliptical integral solution for the large amplitude free vibration of a rectangular single mode plate backed by a multi-acoustic mode cavity. *Thin-Walled Structures* 49(9), 1191–1194 (2011)

[28] Lee Y.Y.: Nonlinear structure-extended cavity interaction simulation using a new version of harmonic balance method. *PLOS ONE* 13(7) Article Number: e0199159 (2018) 10.1371/journal.pone.0199159 29969458 PMC6029766

[29] Younesian D., Sadri M., Esmailzadeh E.: Primary and secondary resonance analyses of clamped-clamped micro-beams. *Nonlinear Dynamics*. 76(4), 1867–1884 (2014)

[30] Mangussi F. and Zanette D.H. Internal resonance in a vibrating beam: a zoo of nonlinear resonance peaks. *PLOS ONE* 11(9), Article Number: e0162365 (2016) 10.1371/journal.pone.0162365 27648829 PMC5029881

[31] Leung A.Y.T., Guo Z.: Feed forward residue harmonic balance method for a quadratic nonlinear oscillator. *International Journal of Bifurcation and Chaos* 21(6), 1783–1794(2011)

[32] Xu L.: Dynamics of two-strand yarn spinning in forced vibration. *Nonlinear Analysis-Theory Methods & Applications*. 71(12), 827–829 (2009)

[33] Xu L., Sun L.: Electromechanical coupled non-linear vibration of the micro- plate. *Proceedings of the Institution of Mechanical Engineers*, *Part C*: *Journal of Mechanical Engineering Science* 224, 1383–1396 (2010)

[34] Huang J.L., Su R.K.L., Chen S.H.: Precise Hsu's method for analyzing the stability of periodic solutions of multi-degrees-of-freedom systems with cubic nonlinearity. *Computers & Structures* 87(23–24), 1624–1630 (2009)

[35] Chen S.H., Huang J.L., Sze K.Y.: Multidimensional Lindstedt-Poincare method for nonlinear vibration of axially moving beams. *Journal of Sound and Vibration* 306(1–2), 1–11 (2007)

[36] Guo Z.J., Leung A.Y.T., Ma X.Y.: Solution procedure of residue harmonic balance method and its applications. *Science China-Physics Mechanics & Astronomy* 57(8), 1581–1591 (2014)

[37] Hasan A.S.M.Z., Lee Y.Y., Leung A.Y.T.: The multi-level residue harmonic balance solutions of multi-mode nonlinearly vibrating beams on an elastic foundation. *Journal of Vibration and Control*. 22(14), 3218–3235 10.1177/1077546314562225 (2016)

[38] Fahy F.: *Sound and Structural Vibration*, *Radiation*, *Transmission and Response*. Academic Press 6^th^ edition (2000)

